# Robustness and applicability of transcription factor and pathway analysis tools on single-cell RNA-seq data

**DOI:** 10.1186/s13059-020-1949-z

**Published:** 2020-02-12

**Authors:** Christian H. Holland, Jovan Tanevski, Javier Perales-Patón, Jan Gleixner, Manu P. Kumar, Elisabetta Mereu, Brian A. Joughin, Oliver Stegle, Douglas A. Lauffenburger, Holger Heyn, Bence Szalai, Julio Saez-Rodriguez

**Affiliations:** 1 Institute for Computational Biomedicine, Bioquant, Heidelberg University, Faculty of Medicine, and Heidelberg University Hospital, Heidelberg, Germany; 2 Joint Research Centre for Computational Biomedicine (JRC-COMBINE), RWTH Aachen University, Faculty of Medicine, Aachen, Germany; 3 Department of Knowledge Technologies, Jožef Stefan Institute, Ljubljana, Slovenia; 4 German Cancer Research Center (DKFZ), Heidelberg, Germany; 5 European Molecular Biology Laboratory (EMBL), Genome Biology Unit, Heidelberg, Germany; 6 Department of Biological Engineering, MIT, Cambridge, MA USA; 7 CNAG-CRG, Centre for Genomic Regulation (CRG), Barcelona Institute of Science and Technology (BIST), Barcelona, Spain; 8 Koch Institute for Integrative Cancer Biology, MIT, Cambridge, MA USA; 9 European Molecular Biology Laboratory, European Bioinformatics Institute, Wellcome Genome Campus, Cambridge, UK; 10 Universitat Pompeu Fabra (UPF), Barcelona, Spain; 11 Faculty of Medicine, Department of Physiology, Semmelweis University, Budapest, Hungary

**Keywords:** scRNA-seq, Functional analysis, Transcription factor analysis, Pathway analysis, Benchmark

## Abstract

**Background:**

Many functional analysis tools have been developed to extract functional and mechanistic insight from bulk transcriptome data. With the advent of single-cell RNA sequencing (scRNA-seq), it is in principle possible to do such an analysis for single cells. However, scRNA-seq data has characteristics such as drop-out events and low library sizes. It is thus not clear if functional TF and pathway analysis tools established for bulk sequencing can be applied to scRNA-seq in a meaningful way.

**Results:**

To address this question, we perform benchmark studies on simulated and real scRNA-seq data. We include the bulk-RNA tools PROGENy, GO enrichment, and DoRothEA that estimate pathway and transcription factor (TF) activities, respectively, and compare them against the tools SCENIC/AUCell and metaVIPER, designed for scRNA-seq. For the in silico study, we simulate single cells from TF/pathway perturbation bulk RNA-seq experiments. We complement the simulated data with real scRNA-seq data upon CRISPR-mediated knock-out. Our benchmarks on simulated and real data reveal comparable performance to the original bulk data. Additionally, we show that the TF and pathway activities preserve cell type-specific variability by analyzing a mixture sample sequenced with 13 scRNA-seq protocols. We also provide the benchmark data for further use by the community.

**Conclusions:**

Our analyses suggest that bulk-based functional analysis tools that use manually curated footprint gene sets can be applied to scRNA-seq data, partially outperforming dedicated single-cell tools. Furthermore, we find that the performance of functional analysis tools is more sensitive to the gene sets than to the statistic used.

## Background

Gene expression profiles provide a blueprint of the status of cells. Thanks to diverse high-throughput techniques, such as microarrays and RNA-seq, expression profiles can be collected relatively easily and are hence very common. To extract functional and mechanistic information from these profiles, many tools have been developed that can, for example, estimate the status of molecular processes such as the activity of pathways or transcription factors (TFs). These functional analysis tools are broadly used and belong to the standard toolkit to analyze expression data [1–4].

Functional analysis tools typically combine prior knowledge with a statistical method to gain functional and mechanistic insights from omics data. In the case of transcriptomics, prior knowledge is typically rendered as gene sets containing genes belonging to, e.g., the same biological process or to the same Gene Ontology (GO) annotation. The Molecular Signature Database (MSigDB) is one of the largest collections of curated and annotated gene sets [5]. Statistical methods are as abundant as the different types of gene sets. Among them, the most commonly used are over-representation analysis (ORA) [6] and Gene Set Enrichment Analysis (GSEA) [7]. Still, there is a growing number of statistical methods spanning from simple linear models to advanced machine learning methods [8, 9].

Recent technological advances in single-cell RNA-seq (scRNA-seq) enable the profiling of gene expression at the individual cell level [10]. Multiple technologies and protocols have been developed, and they have experienced a dramatic improvement over recent years. However, single-cell data sets have a number of limitations and biases, including low library size and drop-outs. Bulk RNA-seq tools that focus on cell type identification and characterization as well as on inferring regulatory networks can be readily applied to scRNA-seq data [11]. This suggests that functional analysis tools should in principle be applicable to scRNA-seq data as well. However, it has not been investigated yet whether these limitations could distort and confound the results, rendering the tools not applicable to single-cell data.

In this paper, we benchmarked the robustness and applicability of various TF and pathway analysis tools on simulated and real scRNA-seq data. We focused on three tools for bulk and three tools for scRNA-seq data. The bulk tools were PROGENy [12], DoRothEA [13], and classical GO enrichment analysis, combining GO gene sets [14] with GSEA. PROGENy estimates the activity of 14 signaling pathways by combining corresponding gene sets with a linear model. DoRothEA is a collection of resources of TF’s targets (regulons) that can serve as gene sets for TF activity inference. For this study, we coupled DoRothEA with the method VIPER [15] as it incorporates the mode of regulation of each TF-target interaction. Both PROGENy’s and DoRothEA’s gene sets are based on observing the transcriptomic consequences (the “footprint”) of the processes of interest rather than the genes composing the process as gene sets [16]. This approach has been shown to be more accurate and informative in inferring the process’s activity [12, 17]. The tools specifically designed for application on scRNA-seq data that we considered are SCENIC/AUCell [18] and metaVIPER [19]. SCENIC is a computational workflow that comprises the construction of gene regulatory networks (GRNs) from scRNA-seq data that are subsequently interrogated to infer TF activity with the statistical method AUCell. In addition, we coupled AUCell with the footprint-based gene sets from DoRothEA and PROGENy that we hereafter refer to as D-AUCell and P-AUCell. Using DoRothEA with both VIPER and AUCell on scRNA-seq for TF activity inference allowed us to compare the underlying statistical methods more objectively. metaVIPER is an extension of VIPER which is based on the same statistical method but relies on multiple GRNs such as tissue-specific networks.

We first benchmarked the tools on simulated single-cell transcriptome profiles. We found that on this in silico data the footprint-based gene sets from DoRothEA and PROGENy can functionally characterize simulated single cells. We observed that the performance of the different tools is dependent on the used statistical method and properties of the data, such as library size. We then used real scRNA-seq data upon CRISPR-mediated knock-out/knock-down of TFs [20, 21] to assess the performance of TF analysis tools. The results of this benchmark further supported our finding that TF analysis tools can provide accurate mechanistic insights into single cells. Finally, we demonstrated the utility of the tools for pathway and TF activity estimation on recently published data profiling a complex sample with 13 different scRNA-seq technologies [22]. Here, we showed that summarizing gene expression into TF and pathway activities preserves cell-type-specific information and leads to biologically interpretable results. Collectively, our results suggest that the bulk- and footprint-based TF and pathway analysis tools DoRothEA and PROGENy partially outperform the single-cell tools SCENIC, AUCell, and metaVIPER. Although on scRNA-seq data DoRothEA and PROGENy were less accurate than on bulk RNA-seq, we were still able to extract relevant functional insight from scRNA-seq data.

## Results

### Robustness of bulk-based TF and pathway analysis tools against low gene coverage

Single-cell RNA-seq profiling is hampered by low gene coverage due to drop-out events [23]. In our first analysis, we focused solely on the low gene coverage aspect and whether tools designed for bulk RNA-seq can deal with it. Specifically, we aimed to explore how DoRothEA, PROGENy, and GO gene sets combined with GSEA (GO-GSEA) can handle low gene coverage in general, independently of other technical artifacts and characteristics from scRNA-seq protocols. Thus, we conducted this benchmark using bulk transcriptome benchmark data. In these studies, single TFs and pathways are perturbed experimentally, and the transcriptome profile is measured before and after the perturbation. These experiments can be used to benchmark tools for TF/pathway activity estimation, as they should estimate correctly the change in the perturbed TF or pathway. The use of these datasets allowed us to systematically control the gene coverage (see the “Methods” section). The workflow consisted of four steps (Additional file 1: Figure S1a). In the first step, we summarized all perturbation experiments into a matrix of contrasts (with genes in rows and contrasts in columns) by differential gene expression analysis. Subsequently, we randomly replaced, independently for each contrast, logFC values with 0 so that we obtain a predefined number of “covered” genes with a logFC unequal to zero. Accordingly, a gene with a logFC equal to 0 was considered as missing/not covered. Then, we applied DoRothEA, PROGENy, and GO-GSEA to the contrast matrix, subsetted only to those experiments which are suitable for the corresponding tool: TF perturbation for DoRothEA and pathway perturbation for PROGENy and GO-GSEA. We finally evaluate the global performance of the methods with receiver operating characteristic (ROC) and precision-recall (PR) curves (see the “Methods” section). This process was repeated 25 times to account for stochasticity effects during inserting zeros in the contrast matrix (see the “Methods” section).

**Fig. 1:**
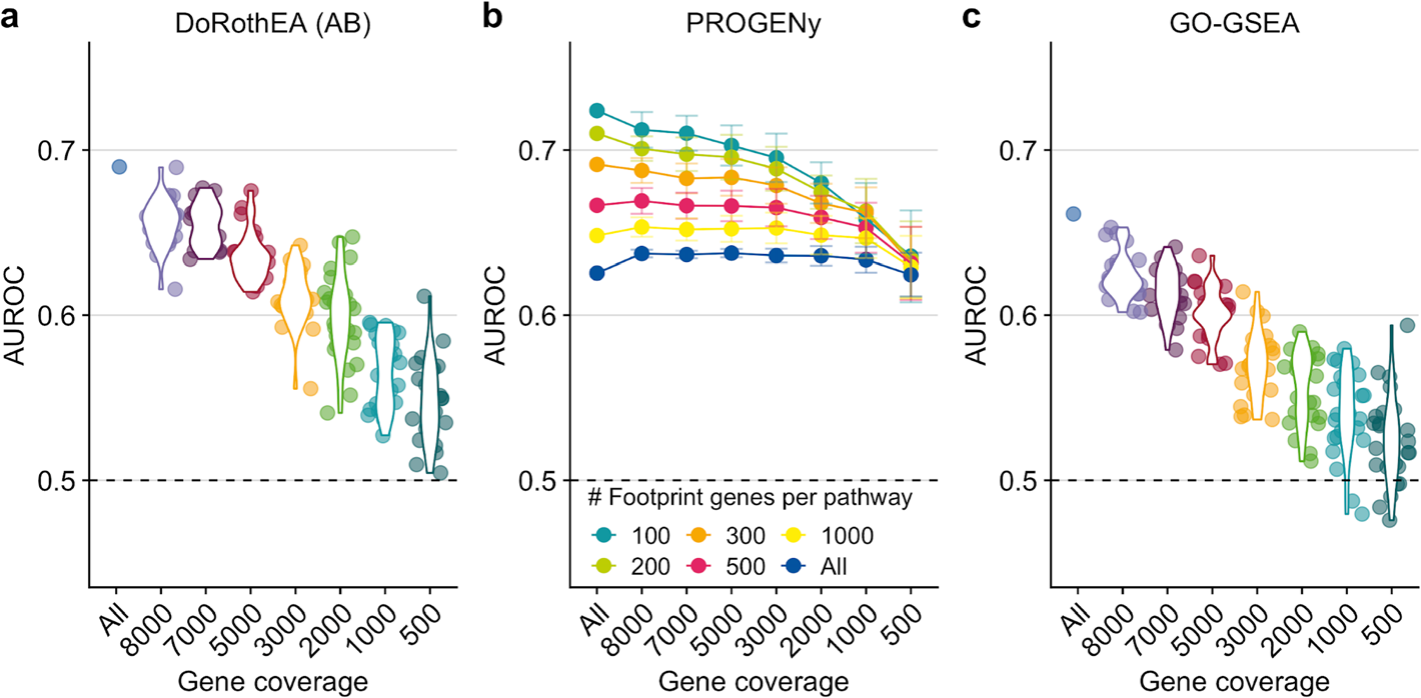
Testing the robustness of DoRothEA (AB), PROGENy, and GO-GSEA against low gene coverage. **a** DoRothEA (AB) performance (area under ROC curve, AUROC) versus gene coverage. **b** PROGENy performance (AUROC) for different number of footprint genes per pathway versus gene coverage. **c** Performance (AUROC) of GO-GSEA versus gene coverage. The dashed line indicates the performance of a random model. The colors in **a** and **c** are meant only as a visual support to distinguish between the individual violin plots and jittered points

DoRothEA’s TFs are accompanied by an empirical confidence level indicating the confidence in their regulons, ranging from A (most confident) to E (less confident; see the “Methods” section). For this benchmark, we included only TFs with confidence levels A and B (denoted as DoRothEA (AB)) as this combination has a reasonable tradeoff between TF coverage and performance [13]. In general, the performance of DoRothEA dropped as gene coverage decreased. While it showed reasonable prediction power with all available genes (AUROC of 0.690), it approached almost the performance of a random model (AUROC of 0.5) when only 500 genes were covered (mean AUROC of 0.547, Fig. 1a, and similar trend with AUPRC, Additional file 1: Figure S1b).

We next benchmarked pathway activities estimated by PROGENy and GO-GSEA. In the original PROGENy framework, 100 footprint genes are used per pathway to compute pathway activities by default, as it has been shown that this leads to the best performance on bulk samples [12]. However, one can extend the footprint size to cover more genes of the expression profiles. We reasoned that this might counteract low gene coverage and implemented accordingly different PROGENy versions (see the “Methods” section). With the default PROGENy version (100 footprint genes per pathway), we observed a clear drop in the global performance with decreasing gene coverage, even though less drastic than for DoRothEA (from AUROC of 0.724 to 0.636, Fig. 1b, similar trends with AUPRC, Additional file 1: Figure S1c). As expected, PROGENy performed the best with 100 footprint genes per pathway when there is complete gene coverage. The performance differences between the various PROGENy versions shrank with decreasing gene coverage. This suggests that increasing the number of footprint genes can help to counteract low gene coverage. To provide a fair comparison between PROGENy and GO-GSEA, we used only those 14 GO terms that match the 14 PROGENy pathways (Additional file 1: Figure S1d). In general, GO-GSEA showed weaker performance than PROGENy. The decrease in performance was more prominent as gene coverage decreased (from AUROC of 0.662 to 0.525, Fig. 1c, and similar trend with AUPRC, Additional file 1: Figure S1e). With a gene coverage of less than 2000 genes, GO-GSEA performance was no better than random.

As our benchmark data set comprises multiple perturbation experiments per pathway, we also evaluated the performance of PROGENy and GO-GSEA at the pathway level (Additional file 1: Figure S2a and b). The pathway-wise evaluation supported our finding that PROGENy outperforms GO-GSEA across all gene coverages, but the performance between pathways is variable.

In summary, this first benchmark provided insight into the general robustness of the bulk-based tools DoRothEA, PROGENy, and GO-GSEA with respect to low gene coverage. DoRothEA performed reasonably well down to a gene coverage of 2000 genes. The performance of all different PROGENy versions was robust across the entire gene coverage range tested. GO-GSEA showed a worse performance than PROGENy, especially in the low gene coverage range. Since DoRothEA and PROGENy showed promising performance in low gene coverage ranges, we decided to explore them on scRNA-seq data. Due to its poor performance, we did not include GO-GSEA in the subsequent analyses.

### Benchmark on simulated single-cell RNA-seq data

For the following analyses, we expanded the set of tools with the statistical methods AUCell that we decoupled from the SCENIC workflow [18] and metaVIPER [19]. Both methods were developed specifically for scRNA-seq analysis and thus allow the comparison of bulk vs single-cell based tools on scRNA-seq data. AUCell is a statistical method that is originally used with GRNs constructed by SCENIC and assesses whether gene sets are enriched in the top quantile of a ranked gene signature (see the “Methods” section). In this study, we combined AUCell with DoRothEA’s and PROGENy’s gene sets (referred to as D-AUCell and P-AUCell, respectively). metaVIPER is an extension of VIPER and requires multiple gene regulatory networks instead of a single network. In our study, we coupled 27 tissue-specific gene regulatory networks with metaVIPER, which provides a single TF consensus activity score estimated across all networks (see the “Methods” section). To benchmark all these methods on single cells, ideally, we would have scRNA-seq datasets after perturbations of TFs and pathways. However, these datasets, especially for pathways, are currently very rare. To perform a comprehensive benchmark study, we developed a strategy to simulate samples of single cells using bulk RNA-seq samples from TF and pathway perturbation experiments.

A major cause of drop-outs in single-cell experiments is the abundance of transcripts in the process of reverse-transcription of mRNA to cDNA [23]. Thus, our simulation strategy was based on the assumption that genes with low expression are more likely to result in drop-out events.

**Fig. 2:**
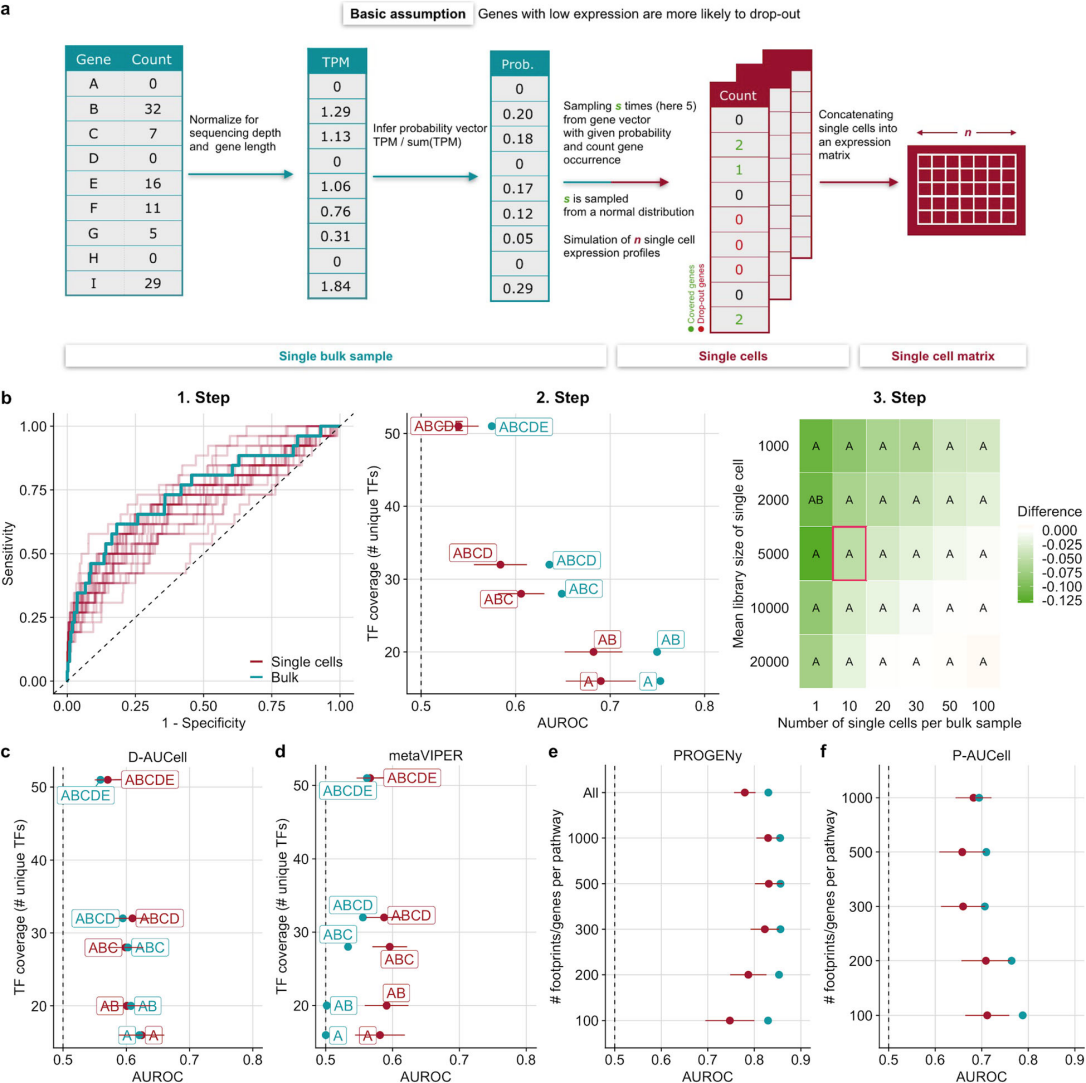
Benchmark results of TF and pathway analysis tools on simulated scRNA-seq data. **a** Simulation strategy of single cells from an RNA-seq bulk sample. **b** Example workflow of DoRothEA’s performance evaluation on simulated single cells for a specific parameter combination (number of cells = 10, mean library size = 5000). 1. Step: ROC-curves of DoRothEA’s performance on single cells (25 replicates) and on bulk data including only TFs with confidence level A. 2. Step: DoRothEA performance on single cells and bulk data summarized as AUROC vs TF coverage. TF coverage denotes the number of distinct perturbed TFs in the benchmark dataset that are also covered by the gene set resource (see Additional file 1: Figure S3a) Results are provided for different combinations of DoRothEA’s confidence levels (A, B, C, D, E). Error bars of AUROC values depict the standard deviation and correspond to different simulation replicates. Step 3: Averaged difference across all confidence level combinations between AUROC of single cells and bulk data for all possible parameter combinations. The letters within the tiles indicates which confidence level combination performs the best on single cells. The tile marked in red corresponds to the parameter setting used for previous plots (Steps 1 and 2). **c** D-AUCell and **d** metaVIPER performance on simulated single cells summarized as AUROC for a specific parameter combination (number of cells = 10, mean library size = 5000) and corresponding bulk data vs TF coverage. **e**, **f** Performance results of **e** PROGENy and **f** P-AUCell on simulated single cells for a specific parameter combination (number of cells = 10, mean library size = 5000) and corresponding bulk data in ROC space vs number of footprint genes per pathway. **c**–**f** Plots revealing the change in performance for all possible parameter combinations (Step 3) are available in Additional file 1: Figure S7. **b**–**f** The dashed line indicates the performance of a random model

The simulation workflow started by transforming read counts of a single bulk RNA-seq sample to transcripts per million (TPM), normalizing for gene length and library size. Subsequently, for each gene, we assigned a sampling probability by dividing the individual TPM values with the sum of all TPM values. These probabilities are proportional to the likelihood for a given gene not to “drop-out” when simulating a single cell from the bulk sample. We determined the total number of gene counts for a simulated single cell by sampling from a normal distribution with a mean equal to the desired library size which is specified as the first parameter of the simulation. We refer hereafter to this number as the library size. For every single cell, we then sampled with replacement genes from the gene probability vector up to the determined library size. The frequency of occurrence of individual genes becomes the new gene count in the single cell. The number of simulated single cells from a single bulk sample can be specified as the second parameter of the simulation. Of note, this parameter is not meant to reflect a realistic number of cells, but it is rather used to investigate the loss of information: the lower the number of simulated cells, the more information is lost from the original bulk sample (Fig. 2a; see the “Methods” section). This simple workflow guaranteed that the information of the original bulk perturbation is preserved and scRNA-seq characteristics, such as drop-outs, low library size, and a high number of samples/cells are introduced.

Our bulk RNA-seq samples comprised 97 single TF perturbation experiments targeting 52 distinct TFs and 15 single pathway perturbation experiments targeting 7 distinct pathways (Additional file 1: Figure S3a and b; see the “Methods” section). We repeated the simulation of single cells from each bulk sample template to account for the stochasticity of the simulation procedure. We tested our simulation strategy by comparing the characteristics of the simulated cells to real single cells. In this respect, we compared the count distribution (Additional file 1: Figure S4a), the relationship of mean and variance of gene expression (Additional file 1: Figure S4b), and the relationship of library size to the number of detected genes (Additional file 1: Figure S4c). These comparisons suggested that our simulated single cells closely resemble real single cells and are thus suitable for benchmarking.

Unlike in our first benchmark, we applied the TF and pathway analysis tools directly on single samples/cells and built the contrasts between perturbed and control samples at the level of pathway and TF activities (see the “Methods” section). We compared the performance of all tools to recover the perturbed TFs/pathways. We also considered the performance on the template bulk data, especially for the bulk-based tools DoRothEA and PROGENy, as a baseline for comparison to their respective performance on the single-cell data.

We show, as an example, the workflow of the performance evaluation for DoRothEA (Fig. 2b, 1. Step). As a first step, we applied DoRothEA to single cells generated for one specific parameter combination and bulk samples, performed differential activity analysis (see the “Methods” section), and evaluated the performance with ROC and PR curves including only TFs with confidence level A. In this example, we set the number of cells to 10 as this reflects an observable loss of information of the original bulk sample and the mean library size to 5000 as this corresponds to a very low but still realistic sequencing depths of scRNA-seq experiments. Each repetition of the simulation is depicted by an individual ROC curve, which shows the variance in the performance of DoRothEA on simulated single-cell data (Fig. 2b, 1. Step). The variance decreases as the library size and the number of cells increase (which holds true for all tested tools, Additional file 1: Figure S5a–e). The shown ROC curves are summarized into a single AUROC value for bulk and mean AUROC value for single cells. We performed this procedure also for different TF confidence level combinations and show the performance change in these values in relation to the number of distinct perturbed TFs in the benchmark that are also covered by the gene set resources that we refer to as TF coverage (Fig. 2b, 2. Step). For both bulk and single cells, we observe a tradeoff between TF coverage and performance caused by including different TF confidence level combinations in the benchmark. This result is supported by both AUROC and AUPRC (Additional file 1: Figure S6a) and corresponds to our previous findings [13]. The performance of DoRothEA on single cells does not reach the performance on bulk, though it can still recover TF perturbations on the simulated single cells reasonably well. This is especially evident for the most confident TFs (AUROC of 0.690 for confidence level A and 0.682 for the confidence level combination AB). Finally, we explore the effect of the simulation parameters library size and the number of cells on the performance by performing the previously described analysis for all combinations of library sizes and cell numbers. We computed the mean difference between AUROC scores of single-cell and bulk data across all confidence level combinations. A negative difference indicates that the tool of interest performs overall better on bulk data than on scRNA-seq data, and a positive difference that it performs better on scRNA-seq. We observed a gradually decreasing negative difference approaching 0 when the size of the library and the number of cells increase (Fig. 2b, 3. Step, and Additional file 1: Figure S7a). Note, however, that the number of cells and thus the amount of lost information of the original bulk sample has a stronger impact on the performance than the mean library size. In addition, we identified the best performing combination of DoRothEA’s TF confidence levels for different library sizes and the number of single cells. Thus, the results can be used as recommendations for choosing the confidence levels on data from an experiment with comparable characteristics in terms of sequencing depths.

Similarly to DoRothEA, we also observed for D-AUCell a tradeoff between TF coverage and performance on both single cells and bulk samples when using the same parameter combination as before (Fig. 2c, similar trend with AUPRC Additional file 1: Figure S6b). The summarized performance across all confidence level combinations of D-AUCell on single cells slightly outperformed its performance on bulk samples (AUROC of 0.601 on single cells and 0.597 on bulk). This trend becomes more evident with increasing library size and the number of cells (Additional file 1: Figure S7b).

For the benchmark of metaVIPER, we assigned confidence levels to the tissue-specific GTEx regulons based on DoRothEA’s gene set classification. This was done for consistency with DoRothEA and D-AUCell, even if there is no difference in confidence among them. Hence, for metaVIPER, we do not observe a tradeoff between TF coverage and performance (Fig. 2d, similar trend with AUPRC Additional file 1: Figure S6c). As opposed to D-AUCell, metaVIPER performed clearly better on single cells than on bulk samples across all confidence level combinations (AUROC of 0.584 on single cells and 0.531 on bulk). This trend increased with increasing library size and number of cells (Additional file 1: Figure S7c). However, the overall performance of metaVIPER is worse than the performance of DoRothEA and D-AUCell. In summary, the bulk-based tool DoRothEA performed the best on the simulated single cells followed by D-AUCell. metaVIPER performed slightly better than a random model.

For the benchmark of pathway analysis tools, we observed that PROGENy performed well across different number of footprint genes per pathway, with a peak at 500 footprint genes for both single cells and bulk (AUROC of 0.856 for bulk and 0.831 for single cells, Fig. 2e, similar trend with AUPRC Additional file 1: Figure S6d). A better performance for single-cell analysis with more than 100 footprint genes per pathway is in agreement with the previous general robustness study that suggested that a higher number of footprint genes can counteract low gene coverage. Similarly to the benchmark of TF analysis tools, we studied the effect of the simulation parameters on the performance of pathway analysis tools. We averaged for each parameter combination the performance difference between single cells and bulk across the different versions of PROGENy. For the parameter combination associated with Fig. 2e (number of cells = 10, mean library size = 5000), the average distance is negative showing that the performance of PROGENy on bulk was, in general, better than on single-cell data. Increasing the library size and the number of cells improved the performance of PROGENy on single cells reaching almost the same performance as on bulk samples (Additional file 1: Figure S7d). For most parameter combinations, PROGENy with 500 or 1000 footprint genes per pathway yields the best performance.

For P-AUCell, we observed a different pattern than for PROGENy as it worked best with 100 footprint genes per pathway for both single cells and bulk (AUROC of 0.788 for bulk and 0.712 for single cells, Fig. 2f, similar trends with AUPRC Additional file 1: Figure S6e). Similar to PROGENy, increasing the library size and the number of cells improved the performance, but not to the extent of its performance on bulk (Additional file 1: Figure S7e). For most parameter combinations, P-AUCell with 100 or 200 footprint genes per pathway yielded the best performance.

In summary, both PROGENy and P-AUCell performed well on the simulated single cells, and PROGENy performed slightly better. For pathway analysis, P-AUCell did not perform better on scRNA-seq than on bulk data. We then went on to perform a benchmark analysis on real scRNA-seq datasets.

### Benchmark on real single-cell RNA-seq data

After showing that the footprint-based gene sets from DoRothEA and PROGENy can handle low gene coverage and work reasonably well on simulated scRNA-seq data with different statistical methods, we performed a benchmark on real scRNA-seq data. However, single-cell transcriptome profiles of TF and pathway perturbations are very rare. To our knowledge, there are no datasets of pathway perturbations on single-cell level comprehensive enough for a robust benchmark of pathway analysis tools. For tools inferring TF activities, the situation is better: recent studies combined CRISPR knock-outs/knock-down of TFs with scRNA-seq technologies [20, 21] that can serve as potential benchmark data.

The first dataset is based on the Perturb-seq technology, which contains 26 knock-out perturbations targeting 10 distinct TFs after 7 and 13 days of perturbations (Additional file 1: Figure S8a) [20]. To explore the effect of perturbation time, we divided the dataset into two sub-datasets based on perturbation duration (Perturb-seq (7d) and Perturb-seq (13d)). The second dataset is based on CRISPRi protocol and contains 141 perturbation experiments targeting 50 distinct TFs [21] (Additional file 1: Figure S8a). The datasets showed a variation in terms of drop-out rate, the number of cells, and sequencing depths (Additional file 1: Figure S8b).

To exclude bad or unsuccessful perturbations in the case of CRISPRi experiments, we discarded experiments when the logFC of the targeted gene/TF was greater than 0 (12 out of 141, Additional file 1: Figure S8c). This quality control is important only in the case of CRISPRi, as it works on the transcriptional level. Perturb-seq (CRISPR knock-out) acts on the genomic level, so we cannot expect a clear relationship between KO efficacy and transcript level of the target. Note that the logFCs of both Perturb-seq sub-datasets are in a narrower range in comparison to the logFCs of the CRISPRi dataset (Additional file 1: Figure S8d). The perturbation experiments that passed this quality check were used in the following analyses.

We also considered the SCENIC framework for TF analysis [18]. We inferred GRNs for each sub-dataset using this framework (see the “Methods” section). We set out to evaluate the performance of DoRothEA, D-AUCell, metaVIPER, and SCENIC on each benchmark dataset individually.

**Fig. 3:**
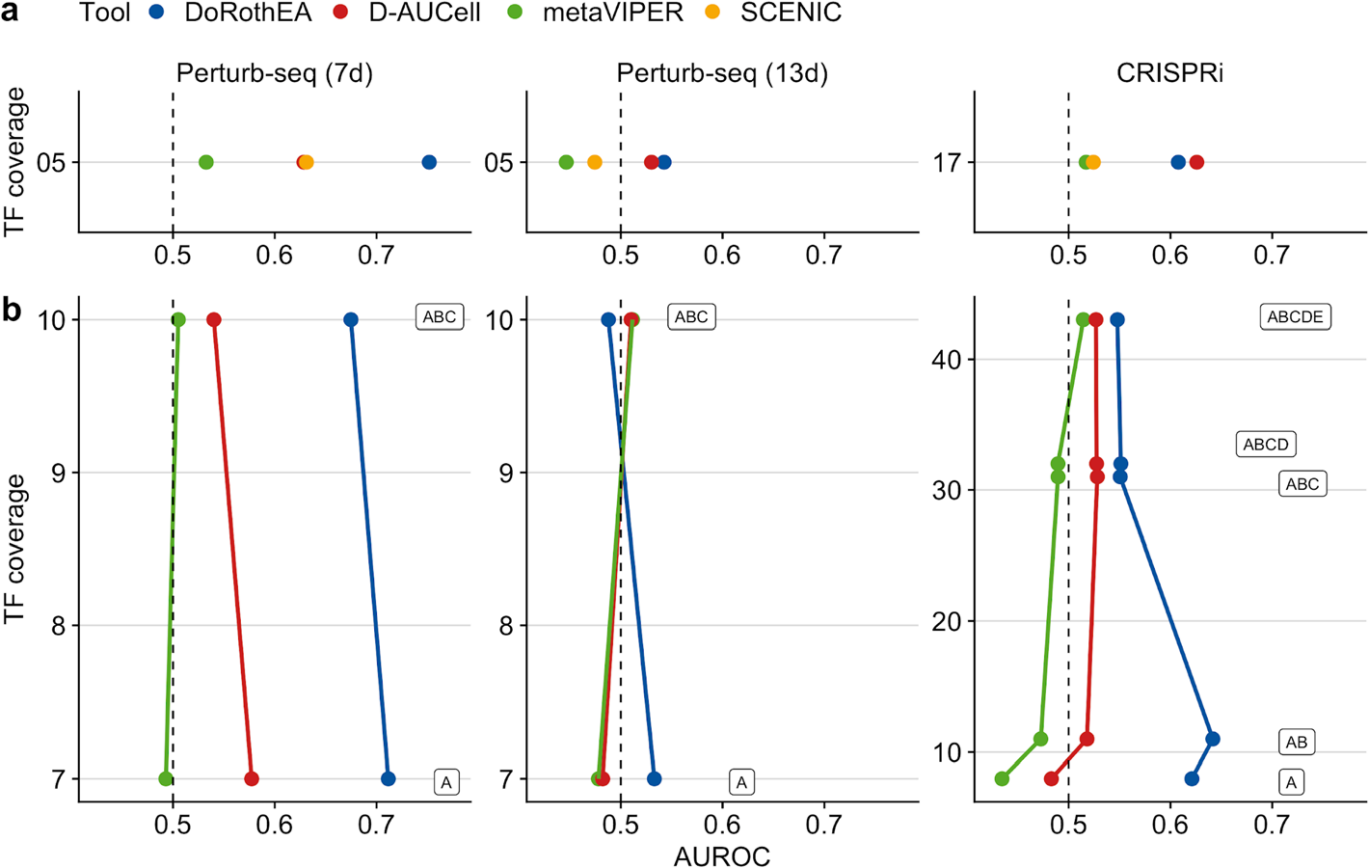
Benchmark results of TF analysis tools on real scRNA-seq data. **a** Performance of DoRothEA, D-AUCell, metaVIPER, and SCENIC on all sub benchmark datasets in ROC space vs TF coverage. **b** Performance of DoRothEA, D-AUCell, and metaVIPER on all sub benchmark datasets in ROC vs TF coverage split up by combinations of DoRothEA’s confidence levels (A-E). **a**, **b** In both panels, the results for each tool are based on the same but for the respective panel different set of (shared) TFs. TF coverage reflects the number of distinct perturbed TFs in the benchmark data set that are also covered by the gene sets

To perform a fair comparison among the tools, we pruned their gene set resources to the same set of TFs. However, the number of TFs in the dataset-specific SCENIC networks was very low (109 for Perturb-Seq (7d), 126 for Perturb-Seq (13d), and 182 TFs for CRISPRi), yielding a low overlap with the other gene set resources. Therefore, only a small fraction of the benchmark dataset was usable yielding low TF coverage. Nevertheless, we found that DoRothEA performed the best on the Perturb-seq (7d) dataset (AUROC of 0.752, Fig. 3a) followed by D-AUCell and SCENIC with almost identical performance (AUROC of 0.629 and 0.631, respectively). metaVIPER performed just slightly better than a random model (AUROC of 0.533). Interestingly, all tools performed poorly on the Perturb-seq (13d) dataset. In the CRISPRi dataset, DoRothEA and D-AUCell performed the best with D-AUCell showing slightly better performance than DoRothEA (AUROC of 0.626 for D-AUCell and 0.608 for DoRothEA). SCENIC and metaVIPER performed slightly better than a random model. Given that we included in this analysis only shared TFs across all gene set resources, we covered only 5 and 17 distinct TFs of the Perturb-seq and CRISPRi benchmark dataset.

To make better use of the benchmark dataset, we repeated the analysis without SCENIC, which resulted in a higher number of shared TFs among the gene set resources and a higher TF coverage. The higher TF coverage allowed us to investigate the performance of the tools in terms of DoRothEA’s confidence level. For both Perturb-seq datasets, we found consistent results with the previous study when the TF coverage increased from 5 to 10 (Fig. 3b). However, for the CRISPRi dataset, the performance of DoRothEA and metaVIPER remained comparable to the previous study while the performance of D-AUCell dropped remarkably. These trends can also be observed in PR-space (Additional file 1: Figure S8e).

In summary, these analyses suggested that the tools DoRothEA and D-AUCell, both interrogating the manually curated, high-quality regulons from DoRothEA, are the best-performing tools to recover TF perturbation at the single-cell level of real data.

### Application of TF and pathway analysis tools on samples of heterogeneous cell type populations (PBMC+HEK293T)

In our last analysis, we wanted to test the performance of all tested tools in a more heterogeneous system that would illustrate a typical scRNA-seq data analysis scenario where multiple cell types are present. We used a dataset from the Human Cell Atlas project [24] that contains scRNA-seq profiles of human peripheral blood mononuclear cells (PBMCs) and HEK 293 T cell line with annotated cell types [22]. This dataset was analyzed with 13 different scRNA-seq protocols (see the “Methods” section). In this study, no ground truth (in contrast to the previous perturbation experiments) for TF and pathway activities was available. To evaluate the performance of all tools, we assessed the potential of TF and pathway activities to cluster cells from the same cell type together based on a priori annotated cell types. All pathway analysis tools and the TF analysis tools DoRothEA, D-AUCell, and metaVIPER were readily applicable to the dataset, except for SCENIC, where we first had to infer GRNs specific for each dataset (and thus experimental protocol) from the respective data (e.g., Drop-seq regulons inferred from the Drop-seq dataset; see the “Methods” section). The overlap of all protocol-specific SCENIC regulons comprised only 24 TFs (Additional file 1: Figure S9a). Including regulons from DoRothEA and GTEx shrank the total overlap down to 20 (Additional file 1: Figure S9b). In contrast, high-quality regulons (confidence levels A and B) from DoRothEA and GTEx alone overlapped in 113 TFs. Given the very low regulon overlap between DoRothEA, GTEx, and all protocol-specific SCENIC regulons, we decided to subset DoRothEA and GTEx to their shared TFs while using all available TFs of the protocol-specific SCENIC regulons.

The low overlap of the SCENIC regulons motivated us to investigate the direct functional consequences of their usage. Theoretically, one would expect to retrieve highly similar regulons as they were constructed from the same biological context. We calculated the pairwise (Pearson) correlations of TF activities between the scRNA-seq technologies for each tool. The distribution of correlation coefficients for each tool denotes the consistency of predicted TF activity across the protocols (Additional file 1: Figure S10). The tools DoRothEA, D-AUCell, and metaVIPER had all a similar median Pearson correlation coefficient of ~ 0.63 and SCENIC of 0.34. This suggests that the predicted TF activities via SCENIC networks are less consistent across the protocols than the TF activities predicted via DoRothEA, D-AUCell, and metaVIPER.

**Fig. 4:**
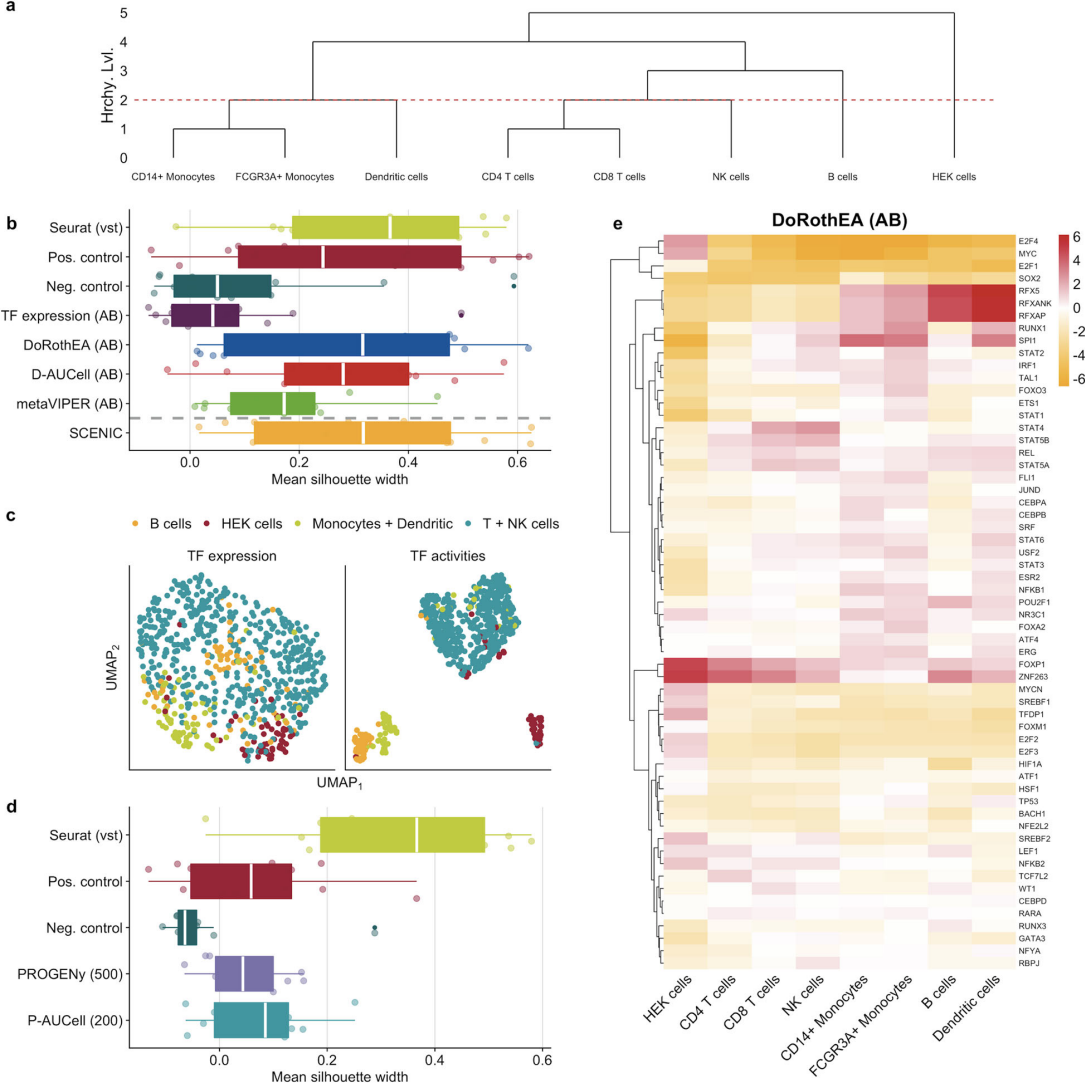
Application of TF and pathway analysis tools on a representative scRNA-seq dataset of PBMCs and HEK cells. **a** Dendrogram showing how cell lines/cell types are clustered together based on different hierarchy levels. The dashed line marks the hierarchy level 2, where CD4 T cells, CD8 T cells, and NK cells are aggregated into a single cluster. Similarly, CD14+ monocytes, FCGR3A+ monocytes, and dendritic cells are also aggregated to a single cluster. The B cells and HEK cells are represented by separate, pure clusters. **b**, **d** Comparison of cluster purity (clusters are defined by hierarchy level 2) between the top 2000 highly variable genes and **b** TF activity and TF expression and **d** pathway activities. The dashed line in **b** separates SCENIC as it is not directly comparable to the other TF analysis tools and controls due to a different number of considered TFs. **c** UMAP plots of TF activities calculated with DoRothEA and corresponding TF expression measured by SMART-Seq2 protocol. **e** Heatmap of selected TF activities inferred with DoRothEA from gene expression data generated via Quartz-Seq2

To assess the clustering capacity of TF and pathway activities, we performed our analysis for each scRNA-seq technology separately to identify protocol-specific and protocol-independent trends. We assumed that the cell-type-specific information should be preserved also on the reduced dimension space of TF and pathway activities if these meaningfully capture the corresponding functional processes. Hence, we assessed how well the individual clusters correspond to the annotated cell types by a two-step approach. First, we applied UMAP on different input matrices, e.g., TF/pathway activities or gene expression, and then we evaluated how well cells from the same cell type cluster together. We considered silhouette widths as a metric of cluster purity (see the “Methods” section). Intuitively, each cell type should form a distinct cluster. However, some cell types are closely related, such as different T cells (CD4 and CD8) or monocytes (CD14+ and FCGR3A+). Thus, we decided to evaluate the cluster purity at different levels of the cell-type hierarchy from fine-grained to coarse-grained. We started with the hierarchy level 0 where every cell type forms a distinct cluster and ended with the hierarchy level 4 where all PBMC cell types and the HEK cell line form a distinct cluster (Fig. 4a). Our main findings rely on hierarchy level 2.

Silhouette widths derived from a set of highly variable genes (HVGs) set the baseline for the silhouette widths derived from pathway/TF activities. We identified the top 2000 HVGs with Seurat [25] using the selection method “vst” as it worked the best in our hands at four out of five hierarchy levels (Additional file 1: Figure S11). For both TF and pathway activity matrices, the number of features available for dimensionality reduction using UMAP was substantially less (113 TFs for DoRothEA/metaVIPER, up to 400 TFs for SCENIC GRNs and 14 pathways, respectively) than for a gene expression matrix containing the top 2000 HVGs. As the number of available features for dimensionality reduction is different between HVGs, TFs, and pathways, we compare the cluster purity among these input features, to a positive and negative control. The positive control is a gene expression matrix with the top *n* HVGs and the negative control is a gene expression matrix with randomly chosen *n* HVGs out of the 2000 HVGs (*n* equals 14 for pathway analysis and 113 for TF analysis). It should be noted that in terms of TF analysis, the positive and negative control is only applicable to DoRothEA, D-AUCell, and metaVIPER as they share the same number of features. As the protocol-specific SCENIC GRNs differ in size (Additional file 1: Figure S9a), each network would require its own positive and negative control.

To evaluate the performance of the TF activity inference methods and the utility of TF activity scores, we determined the cluster purity derived from TF activities predicted by DoRothEA, D-AUCell, metaVIPER, and SCENIC, TF expression, and positive and negative controls. scRNA-seq protocols and input matrices used for dimensionality reduction affected cluster purity significantly (two-way ANOVA *p* values < 2.2e−16 and 4.32e−12, respectively, *p* values and estimations for corresponding linear model coefficients in Additional file 1: Figure S12a; see the “Methods” section). The cluster purity based on TF activities inferred using DoRothEA and D-AUCell did not differ significantly (Fig. 4b, corresponding plots for all hierarchy levels in Additional file 1: Figure S12b). In addition, the cluster purity of both tools was not significantly worse than the purity based on all 2000 HVGs, though we observed a slight trend indicating a better cluster purity based on HVGs. This trend is expected due to the large difference in available features for dimensionality reduction. Instead, a comparison to the positive and negative controls is more appropriate. Both DoRothEA and D-AUCell performed comparably to the positive control but significantly better than the negative control across all scRNA-seq protocols (TukeyHSD post-hoc-test, adj. *p* value of 1.26e−4 for DoRothEA and 7.09e−4 for D-AUCell). The cluster purity derived from metaVIPER was significantly worse than for DoRothEA (TukeyHSD post-hoc-test, adj. *p* value of 0.054) and tend to be worse than D-AUCell (TukeyHSD post-hoc-test, adj. *p* value of 0.163) as well. metaVIPER was not significantly better than the negative control. The cluster purity from SCENIC was significantly better than the negative control (TukeyHSD post-hoc-test, adj. *p* value of 1.11e−6) and comparable to the positive control and thus to DoRothEA and D-AUCell. However, as mentioned above, SCENIC is only partially comparable to the controls and other tools due to the different number of TFs.

Regardless of the underlying TF activity tool, except for metaVIPER, the cluster purity derived from TF activities outperformed significantly the purity derived from TF expression (TukeyHSD post-hoc-test, adj. *p* value of 5.89e−6 for DoRothEA, 3.85−e5 for D-AUCell, and 4.0e−8 for SCENIC). This underlines the advantage and relevance of using TF activities over the expression of the TF itself (Fig. 4c). With a comparable performance to a similar number of HVG and also to 2000 HVGs, we concluded that TF activities serve—independently of the underlying scRNA-seq protocol—as a complementary approach for cluster analysis that is based on generally more interpretable cell type marker.

To evaluate the performance of pathway inference methods and the utility of pathway activity scores, we determined cluster purity with pathway matrices generated by different PROGENy versions and P-AUCell. We used 200 and 500 footprint genes per pathway for PROGENy and P-AUCell, respectively, since they provided the best performance in the previous analyses. As observed already for the TF analysis tools, scRNA-seq protocols and matrices used for dimensionality reduction affected cluster purity significantly (two-way ANOVA *p* values of 2.84e−7 and 1.13e−13, respectively, *p* values and estimations for corresponding linear model coefficients in Additional file 1: Figure S13a; see the “Methods” section). The cluster purity derived from pathway activity matrices is not significantly different between PROGENy and P-AUCell, while worse than all HVGs (TukeyHSD post-hoc-test, adj. *p* value of 4.07e−10 for PROGENy and 4.59e−9 for P-AUCell, Fig. 4d, corresponding plots for all hierarchy levels in Additional file 1: Figure S13b). This is expected due to the large difference in the number of available features for dimensionality reduction (2000 HVGs vs 14 pathways). The cluster purity of both approaches was comparable to the positive control but significantly better than the negative control (TukeyHSD post-hoc-test, adj. *p* value of 0.077 for PROGENy and 0.013 for P-AUCell vs negative control). In summary, this study indicated that the pathway activities contain relevant and cell-type-specific information, even though they do not capture enough functional differences to be used for effective clustering analysis. Overall, the cluster purity of cells represented by the estimated pathway activities is worse than the cluster purity of cells represented by the estimated TF activities.

In addition, we observed that TF and pathway matrices derived from Quartz-Seq2 protocol yielded for hierarchy level 2 in significantly better cluster purity than all other protocols, which is in agreement with the original study of the PBMC + HEK293T data (Additional file 1: Figure S12a and S13a) [22].

TF and pathway activity scores are more interpretable than the expression of single genes. Hence, we were interested to explore whether we could recover known cell-type-specific TF and pathway activities from the PBMC data. We decided to focus on the dataset measured with Quartz-Seq2 as this protocol showed in our and in the original study superior performance over all other protocols [22]. We calculated mean TF and pathway activity scores for each cell type using DoRothEA, D-AUCell, metaVIPER, and SCENIC (using only TFs with confidence levels A and B, Fig. 4e and Additional file 1: Figure S14a–c, respectively), PROGENy with 500 and P-AUCell with 200 footprint genes per pathway (Additional file 1: Figure S14d and e). In terms of TF activities, we observed high RFXAP, RFXANK, and RFX5 activity (TFs responsible for MHCII expression) in monocytes, dendritic cells, and B cells (the main antigen-presenting cells of the investigated population [26]) (Additional file 1: Figure S14a and b). Myeloid lineage-specific SPI1 activity [27] was observed in monocytes and dendritic cells. The high activity of repressor TF (where regulation directionality is important) FOXP1 in T lymphocytes [28] was only revealed by DoRothEA. Proliferative TFs like Myc and E2F4 had also high activity in HEK cells.

Regarding pathway activities, we observed across both methods, in agreement with the literature, high activity of NFkB and TNFa in monocytes [29] and elevated Trail pathway activity in B cells (Additional file 1: Figure S14d and e) [30]. HEK cells, as expected from dividing cell lines, had higher activity of proliferative pathways (MAPK, EGFR, and PI3K, Additional file 1: Figure S14d). These later pathway activity changes were only detected by PROGENy but not with AUCell, highlighting the importance of directionality information.

Besides these individual examples, we analyzed the biological relevance of the identified TF activities in more detail. We assumed that the highly active TFs are regulating important cellular functions, resulting in a correlation between TF activity and essentiality. As (to our knowledge) no gene essentiality data is available for PBMCs, we used hematologic cancer (lymphoma and leukemia) gene essentiality data from the DepMap project [31]. We compared the difference between the TF activities in lymphoid (B, T, and NK cells) and myeloid (monocytes and dendritic cells) PBMCs with the TF gene essentiality differences between myeloid and lymphoid hematologic cancers. SPI1, according to its higher activity in myeloid PBMCs, was more essential in myeloid leukemias (Additional file 1: Figure S15a and b, Wilcoxon-test *p* value = 0.038). For a more comprehensive analysis, we compared the differences in TF activity (PBMCs, lymphoid - myeloid) and the differences in TF gene essentiality (hematologic cancers, lymphoid - myeloid) by calculating their Pearson correlation for all TFs. The TF activities predicted by DoRothEA correlated best with respective essentiality scores across all scRNA-seq protocols (median Pearson correlation coefficient of 0.107; 0.08 for D-AUCell; 0.04 for metaVIPER; and − 0.002 for SCENIC, Additional file 1: Figure S15c). The difference in TF activities predicted with DoRothEA from the dataset generated by Smart-Seq2 and Quartz-Seq2 correlated significantly with the difference in essentiality (Pearson correlation, *p* value of 0.049 and 0.032, respectively). Thus, TF activities predicted with DoRothEA regulons correlate, albeit, weakly with gene/TF essentiality.

In summary, the analysis of this mixture sample demonstrated that summarizing gene expression into TF activities can preserve cell type-specific information while drastically reducing the number of features. Hence, TF activities could be considered as an alternative to gene expression for clustering analysis. Furthermore, they correlate, albeit weakly, with gene/TF essentiality, suggesting the biological relevance of the identified cell-type-specific TF activities.

We also showed that pathway activity matrices contain cell-type-specific information, too, although we do not recommend using them for clustering analysis as the number of features is too low. In addition, we recovered known pathway/TF cell-type associations showing the importance of directionality and supporting the utility and power of the functional analysis tools DoRothEA and PROGENy.

## Discussion

In this paper, we tested the robustness and applicability of functional analysis tools on scRNA-seq data. We included both bulk- and single-cell-based tools that estimate either TF or pathway activities from gene expression data and for which well-defined benchmark data exist. The bulk-based tools were DoRothEA, PROGENy, and GO gene sets analyzed with GSEA (GO-GSEA). The functional analysis tools specifically designed for the application in single cells were SCENIC, AUCell combined with DoRothEA (D-AUCell) and PROGENy (P-AUCell) gene sets, and metaVIPER.

We first explored the effect of low gene coverage in bulk data on the performance of the bulk-based tools DoRothEA, PROGENy, and GO-GSEA. We found that for all tools the performance dropped with decreasing gene coverage but at a different rate. While PROGENy was robust down to 500 covered genes, DoRothEA’s performance dropped markedly at 2000 covered genes. In addition, the results related to PROGENy suggested that increasing the number of footprint genes per pathway counteracted low gene coverage. GO-GSEA showed the strongest drop and did not perform better than a random guess below 2000 covered genes. Comparing the global performance across all pathways of both pathway analysis tools suggests that footprint-based gene sets are superior over gene sets containing pathway members (e.g., GO gene sets) in recovering perturbed pathways. This observation is in agreement with previous studies conducted by us and others [12, 32]. However, both PROGENy and GO-GSEA performed poorly for some pathways, e.g., WNT pathway. We reason that this observation might be due to the quality of the corresponding benchmark data [33]. Given this fact and that GO-GSEA cannot handle low gene coverage (in our hands), we concluded that this approach is not suitable for scRNA-seq analysis. Hence, we decided to focus only on PROGENy as bulk-based pathway analysis tool for the following analyses.

Afterward, we benchmarked DoRothEA, PROGENy, D-AUCell, P-AUCell, and metaVIPER on simulated single cells that we sampled from bulk pathway/TF perturbation samples. We showed that our simulated single cells possess characteristics comparable to real single-cell data, supporting the relevance of this strategy. Different combinations of simulation parameters can be related to different scRNA-seq technologies. For each combination, we provide a recommendation of how to use DoRothEA’s and PROGENy’s gene sets (in terms of confidence level combination or number of footprint genes per pathway) to yield the best performance. It should be noted that our simulation approach, as it is now, allows only the simulation of a homogenous cell population. This would correspond to a single cell experiment where the transcriptome of a cell line is profiled. In future work, this simulation strategy could be adapted to account for a heterogeneous dataset that would resemble more realistic single-cell datasets [34, 35].

In terms of TF activity inference, DoRothEA performed best on the simulated single cells followed by D-AUCell and then metaVIPER. Both DoRothEA and D-AUCell shared DoRothEA’s gene set collection but applied different statistics. Thus, we concluded that, in our data, VIPER is more suitable to analyze scRNA-seq data than AUCell. The tool metaVIPER performed only slightly better than a random model, and since it uses VIPER like DoRothEA, the weak performance must be caused by the selection of the gene set resource. DoRothEA’s gene sets/TF regulons were constructed by integrating different types of evidence spanning from literature curated to predicted TF-target interactions. For metaVIPER, we used 27 tissue-specific GRNs constructed in a data-driven manner with ARACNe [36] thus containing only predicted TF-target interactions. The finding that especially the high-confidence TF regulons from DoRothEA outperform pure ARACNe regulons is in agreement with previous observations [13, 37] and emphasizes the importance of combining literature curated resources with in silico predicted resources. Moreover, we hypothesize based on the pairwise comparison that for functional analysis, the choice of gene sets is of higher relevance than the choice of the underlying statistical method.

As one could expect, the single-cell tools D-AUCell metaVIPER performed better on single cells than on the original bulk samples. This trend becomes more pronounced with increasing library size and number of cells. However, the bulk-based tools performed even better on the simulated single cells than the scRNA specific tools.

Related to pathway analysis, both PROGENy and P-AUCell performed well on the simulated single cells. The original framework of PROGENy uses a linear model that incorporates individual weights of the footprint genes, denoting the importance and also the sign of the contribution (positive/negative) to the pathway activity score. Those weights cannot be considered when applying AUCell with PROGENy gene sets. The slightly higher performance of PROGENy suggests that individual weights assigned to gene set members can improve the activity estimation of biological processes.

Subsequently, we aimed to validate the functional analysis tools on real single-cell data. While we could not find suitable benchmark data of pathway perturbations, we exploited two independent datasets of TF perturbations to benchmark the TF analysis tools which we extended with SCENIC. These datasets combined CRISPR-mediated TF knock-out/knock-down (Perturb-Seq and CRISPRi) with scRNA-seq. It should be noted that pooled screenings of gene knock-outs with Perturb-seq suffer from an often faulty assignment of guide-RNA and single-cell [38]. Those mislabeled data confound the benchmark as the ground-truth is not reliable. In addition, our definition of true-positives and true-negatives is commonly used for such analyses [4, 13, 37], but it might be incorrect due to indirect and compensatory mechanisms [39]. These phenomena can confound the results of this type of benchmarks.

Nevertheless, we showed that DoRothEA’s gene sets were globally effective in inferring TF activity from single-cell data with varying performance dependent on the used statistical method. As already shown in the in silico benchmark, D-AUCell showed a weaker performance than DoRothEA, supporting that VIPER performs better than AUCell. Interestingly, metaVIPER’s performance was no better than random across all datasets. metaVIPER used the same statistical method as DoRothEA but different gene set resources. This further supports our hypothesis that the selection of gene sets is more important than the statistical method for functional analysis. This trend is also apparent when comparing the performance of SCENIC and D-AUCell as both rely on the statistical method AUCell but differ in their gene set resource. SCENICs’ performance was consistently weaker than D-AUCell. In addition, we found that the gene regulatory networks inferred with the SCENIC workflow covered only a limited number of TFs in comparison to the relatively comprehensive regulons from DoRothEA or GTEx.

Furthermore, the perturbation time had a profound effect on the performance of the tools: while DoRothEA and D-AUCell worked well for a perturbation duration of 6 (CRISPRi) and 7 days (Perturb-Seq (7d)), the performance dropped markedly for 13 days. We reasoned that, within 13 days of perturbation, compensation effects are taking place at the molecular level that confound the prediction of TF activities. In addition, it is possible that cells without a gene edit outgrow cells with a successful knock-out after 13 days as the knock-out typically yield in a lower fitness and thus proliferation rate.

In summary, DoRothEA subsetted to confidence levels A and B performed the best on real scRNA-seq data but at the cost of the TF coverage. The results of the in silico and in vitro benchmark are in agreement. Accordingly, we believe that it is reasonable to assume that also PROGENy works on real data given the positive benchmark results on simulated data.

Finally, we applied our tools of interest to a mixture sample of PBMCs and HEK cells profiled with 13 different scRNA-seq protocols. We investigated to which extent pathway and TF matrices retain cell-type-specific information, by evaluating how well cells belonging to the same cell type or cell type family cluster together in reduced dimensionality space. Given the lower numbers of features available for dimensionality reduction using TF and pathway activities, cell types could be recovered equally well as when using the same number of the top highly variable genes. In addition, we showed that cell types could be recovered more precisely using TF activities than TF expression, which is in agreement with previous studies [19]. This suggests that summarizing gene expression as TF and pathway activities can lead to noise filtering, particularly relevant for scRNA-seq data, though TF activities performed better than pathway activities which is again attributed to the even lower number of pathways. Specifically, TF activities computed with DoRothEA, D-AUCell, and SCENIC yielded a reasonable cluster purity. It should be noted that, while DoRothEA and D-AUCell rely on independent regulons, the SCENIC networks are constructed from the same dataset they are applied to. This poses the risk of overfitting. Across technologies, the TF activities from SCENIC correlated less well than those calculated with the other tools, which is consistent with overfitting by SCENIC, but further analysis is required.

Our analysis suggested at different points that the performance of TF and pathway analysis tools is more sensitive to the selection of gene sets than the statistical methods. In particular, manually curated footprint gene sets seem to perform generally better. This hypothesis could be tested in the future by decoupling functional analysis tools into gene sets and statistics. Benchmarking all possible combinations of gene sets and statistics (i.e., DoRothEA gene sets with a linear model or PROGENy gene sets with VIPER) would shed light on this question which we believe is of high relevance for the community.

## Conclusions

Our systematic and comprehensive benchmark study suggests that functional analysis tools that rely on manually curated footprint gene sets are effective in inferring TF and pathway activity from scRNA-seq data, partially outperforming tools specifically designed for scRNA-seq analysis. In particular, the performance of DoRothEA and PROGENy was consistently better than all other tools. We showed the limits of both tools with respect to low gene coverage. We also provided recommendations on how to use DoRothEA’s and PROGENy’s gene sets in the best way dependent on the number of cells, reflecting the amount of available information, and sequencing depths. Furthermore, we showed that TF and pathway activities are rich in cell-type-specific information with a reduced amount of noise and provide an intuitive way of interpretation and hypothesis generation. We provide our benchmark data and code to the community for further assessment of methods for functional analysis.

## Methods

### Functional analysis tools, gene set resources, and statistical methods

#### PROGENy

PROGENy is a tool that infers pathway activity for 14 signaling pathways (Androgen, Estrogen, EGFR, Hypoxia, JAK-STAT, MAPK, NFkB, PI3K, p53, TGFb, TNFa, Trail, VEGF, and WNT) from gene expression data [12, 33]. By default pathway activity inference is based on gene sets comprising the top 100 most responsive genes upon corresponding pathway perturbation, which we refer to as footprint genes of a pathway. Each footprint gene is assigned a weight denoting the strength and direction of regulation upon pathway perturbation. Pathway scores are computed by a weighted sum of the product from expression and the weight of footprint genes.

#### DoRothEA

DoRothEA is a gene set resource containing signed transcription factor (TF)-target interactions [13]. Those interactions were curated and collected from different types of evidence such as literature curated resources, ChIP-seq peaks, TF binding site motifs, and interactions inferred directly from gene expression. Based on the number of supporting evidence, each interaction is accompanied by an interaction confidence level ranging from A to E, with A being the most confidence interactions and E the least. In addition, a summary TF confidence level is assigned (also from A to E) which is derived from the leading confidence level of its interactions (e.g., a TF is assigned confidence level A if at least ten targets have confidence level A as well). DoRothEA contains in total 470,711 interactions covering 1396 TFs targeting 20,238 unique genes. We use VIPER in combination with DoRothEA to estimate TF activities from gene expression data, as described in [13].

#### GO-GSEA

We define GO-GSEA as an analysis tool that couples GO-terms from MsigDB with the GSEA framework [7].

#### VIPER

VIPER is a statistical framework that was developed to estimate protein activity from gene expression data using enriched regulon analysis performed by the algorithm aREA [15]. It requires information about interactions (if possible signed) between a protein and its transcriptional targets and the likelihood of their interaction. If not further specified, this likelihood is set to 1. In the original workflow, this regulatory network was inferred from gene expression by the algorithm ARACNe providing mode of regulation and likelihood for each interaction [36]. However, it can be replaced by any other data resource reporting protein target interactions.

#### metaVIPER

metaVIPER is an extension of VIPER that uses multiple gene regulatory networks [19]. TF activities predicted with each individual gene regulatory network are finally integrated to a consensus TF activity score.

#### SCENIC

SCENIC is a computational workflow that predicts TF activities from scRNA-seq data [18]. Instead of interrogating predefined regulons, individual regulons are constructed from the scRNA-seq data. First TF-gene co-expression modules are defined in a data-driven manner with GENIE3. Subsequently, those modules are refined via RcisTarget by keeping only those genes than contain the respective transcription factor binding motif. Once the regulons are constructed, the method AUCell scores individual cells by assessing for each TF separately whether target genes are enriched in the top quantile of the cell signature.

#### D-AUCell/P-AUCell

The statistical method AUCell is not limited to SCENIC regulons. In principle, it can be combined with any gene set resources. Thus, we coupled AUCell with gene sets from DoRothEA (D-AUCell) and PROGENy (P-AUCell). In comparison to other statistical methods, AUCell does not include weights of the gene set members. Thus, the mode of regulation or the likelihood of TF-target interactions or weights of the PROGENy gene sets are not considered for the computation of TF and pathway activities.

### Application of PROGENy on single samples/cells and contrasts

We applied PROGENy on matrices of single samples (genes in rows and either bulk samples or single cells in columns) containing normalized gene expression scores or on contrast matrices (genes in rows and summarized perturbation experiments into contrasts in columns) containing logFCs. In the case of single sample analysis, the contrasts were built based on pathway activity matrices yielding the change in pathway activity (perturbed samples - control sample) summarized as logFC. Independent of the input matrix, we scaled each pathway to have a mean activity of 0 and a standard deviation of 1. We build different PROGENy versions by varying the number of footprint genes per pathway (100, 200, 300, 500, 1000 or all which corresponds to ~ 29,000 genes).

### Application of DoRothEA on single samples/cells and contrasts

We applied DoRothEA in combination with the statistical method VIPER on matrices of single samples (genes in rows and either bulk samples or single cells in columns) containing normalized gene expression scores scaled gene-wise to a mean value of 0 and standard deviation of 1 or on contrast matrices (genes in rows and summarized perturbation experiments into contrasts in columns) containing logFCs. In the case of single sample analysis, the contrasts were built based on TF activity matrices yielding the change in TF activity (perturbed samples - control sample) summarized as logFC. TFs with less than four targets listed in the corresponding gene expression matrix were discarded from the analysis. VIPER provides a normalized enrichment score (NES) for each TF which we consider as a metric for the activity. We used the R package *viper* (version 1.17.0) [15] to run VIPER in combination with DoRothEA.

### Application of GO-GSEA sets on contrasts

We applied GSEA with GO gene sets on contrast matrices (genes in rows and summarized perturbation experiments into contrasts in columns) containing logFCs that serve also as gene-level statistic. We selected only those GO terms which map to PROGENy pathways in order to guarantee a fair comparison between both tools. For the enrichment analysis, we used the R package *fgsea* (version 1.10.0) [40] with 1000 permutations per gene signature.

### Application of metaVIPER on single samples

We ran metaVIPER with 27 tissue-specific gene regulatory networks which we constructed before for one of our previous studies [13]. Those tissue-specific gene regulatory networks were derived using ARACNe [36] taking the database GTEx [41] as tissue-specific gene expression sample resource. We applied metaVIPER on matrices of single samples (genes in rows and single cells in columns) containing normalized gene expression scores scaled gene-wise to a mean value of 0 and a standard deviation of 1. If required, contrasts were built based on TF activity matrices yielding the change in TF activity (perturbed samples - control sample) summarized as logFC. TFs with less than four targets listed in the corresponding input matrix were discarded from the analysis. metaVIPER provides a NES integrated across all regulatory networks for each TF which we consider as a metric for the activity. We used the R package *viper* (version 1.17.0) [15] to run metaVIPER.

### Application of AUCell with either SCENIC, DoRothEA, or PROGENy gene sets on single samples

AUCell is a statistical method to determine specifically for single cells whether a given gene set is enriched at the top quantile of a ranked gene signature. Therefore, AUCell determines the area under the recovery curve to compute the enrichment score. We defined the top quantile as the top 5% of the ranked gene signature. We applied this method coupled with SCENIC, PROGENy, and DoRothEA gene sets. Before applying this method with PROGENy gene sets, we subsetted the footprint gene sets to contain only genes available in the provided gene signature. This guarantees a fair comparison as for the original PROGENy framework with a linear model, the intersection of footprint (gene set) members and signature genes are considered. We applied AUCell with SCENIC, PROGENy, and DoRothEA gene sets on matrices of single samples (genes in rows and single cells in columns) containing raw gene counts. Contrasts were built based on respective TF/pathway activity matrices yielding the change in TF/pathway activity (perturbed samples - control sample) summarized as logFC. For the AUCell analysis, we used the R package *AUCell* (version 1.5.5) [18].

### Induction of artificial low gene coverage in bulk microarray data

We induce the reduction of gene coverage by inserting zeros on the contrast level. In detail, we insert for each contrast separately randomly zeros until we obtained a predefined number of genes with a logFC unequal zero which we consider as “covered”/“measured” genes. We perform this analysis for a gene coverage of 500, 1000, 2000, 3000, 5000, 7000, 8000 and as reference all available genes. To account for stochasticity effects during inserting randomly zero, we repeat this analysis 25 times for each gene coverage value.

### Simulation of single cells

Let C be a vector representing counts per gene for a single bulk sample. C is normalized for gene length and library size resulting in vector B containing TPM values per gene. We assume that samples are obtained from homogenous cell populations and that the probability of a dropout event is inversely proportional to the relative TPM of each measured gene in the bulk sample. Therefore, we define a discrete cumulative distribution function from the vector of gene frequencies $P=\frac{B}{\left|B\right|}$. To simulate a single cell from this distribution, we draw and aggregate L samples by inverse transform sampling. L corresponds to the library size for the count vector of the simulated single cell. We draw L from a normal distribution $N\left(\mu, \frac{\mu }{2}\right)$.

To benchmark the robustness of the methods, we vary the number of cells sampled from a single bulk sample (1, 10, 20, 30, 50, 100) and the value of μ (1000, 2000, 5000, 10.000, 20.000). To account for stochasticity effects during sampling, we repeat this analysis 25 times for each parameter combination.

Prior to normalization, we discarded cells with a library size lower than 100. We normalized the count matrices of the simulated cells by using the R package *scran* (version 1.11.27) [42]. Contrast matrices were constructed by comparing cells originating from one of the perturbation bulk samples vs cells originating from one of the control bulk samples.

### Gene regulatory network (GRN) reconstruction using SCENIC

We infer GRNs on individual sub-datasets using the SCENIC (v. 1.1.2-2) workflow [18]. In brief, gene expression was filtered using default parameters and log2-transformed for co-expression analysis following the recommendations by the authors. We identified potential targets of transcription factors (TFs) based on their co-expression to TFs using GENIE3 (v. 1.6.0, Random Forest with 1000 trees). We pruned co-expression modules to retrieve only putative direct-binding interactions using RcisTarget (v. 1.4.0) and the cis-regulatory DNA-motif databases for hg38 human genome assembly (Version 9 - mc9nr, with distances TSS+/− 10kbp and 500bpUp100Dw, from https://resources.aertslab.org/cistarget/) with default parameters. Only modules with a significant motif enrichment of the TF upstream were kept for the final GRN. While we were running the workflow, 75 genes out of 27,091 from the first DNA-motif database (TSS+/− 10kbp) were inconsistent, i.e., were not described in the second one (500bpUp100Dw), leading to an error of the workflow execution. Thus, these 75 genes were discarded from the database to complete the workflow.

### Benchmarking process with ROC and PR metrics

To transform the benchmark into a binary setup, all activity scores of experiments with negative perturbation effect (inhibition/knockdown) are multiplied by −1. This guarantees that TFs/pathways belong to a binary class either deregulated or not regulated and that the perturbed pathway/TF has in the ideal case the highest activity.

We performed the ROC and PR analysis with the R package *yardstick* (version 0.0.3; https://github.com/tidymodels/yardstick). For the construction of ROC and PR curves, we calculated for each perturbation experiment pathway (or TF) activities. As each perturbation experiment targets either a single pathway (or TF), only the activity score of the perturbed pathway (or TF) is associated with the positive class (e.g., EGFR pathway activity score in an experiment where EGFR was perturbed). Accordingly, the activity scores of all non-perturbed pathways (or TFs) belong to the negative class (e.g., EGFR pathway activity score in an experiment where the JAK-STAT pathway was perturbed). Using these positive and negative classes, Sensitivity/(1-Specificity) or Precision/Recall values were calculated at different thresholds of activity, producing the ROC/PR curves.

### Collecting, curating, and processing of transcriptomic data

#### General robustness study

We extracted single-pathway and single-TF perturbation data profiled with microarrays from a previous study conducted by us [33]. We followed the same procedure of collection, curating, and processing the data as described in the previous study.

#### In silico benchmark

For the simulation of single cells, we collected, curated, and processed single TF and single pathway perturbation data profiled with bulk RNA-seq. We downloaded basic metadata of single TF perturbation experiments from the ChEA3 web-server (https://amp.pharm.mssm.edu/chea3/) [37] and refined the experiment and sample annotation (Additional file 2). Metadata of single pathway perturbation experiments were manually extracted by us from Gene Expression Omnibus (GEO) [43] (Additional file 3). Count matrices for all those experiments were downloaded from ARCHS^4^ (https://amp.pharm.mssm.edu/archs4/) [44].

We normalized count matrices by first calculating normalization factors and second transforming count data to log2 counts per million (CPM) using the R packages *edgeR* (version 3.25.8) [45] and *limma* (version 3.39.18) [46], respectively.

#### In vitro benchmark

To benchmark VIPER on real single-cell data, we inspected related literature and identified two publications which systematically measure the effects of transcription factors on gene expression in single cells:

Dixit et al. introduced Perturb-seq and measured the knockout-effects of ten transcription factors on K562 cells 7 and 13 days after transduction [20]. We downloaded the expression data from GEO (GSM2396858 and GSM2396859) and sgRNA-cell mappings made available by the author upon request in the files promoters_concat_all.csv (for GSM2396858) and pt2_concat_all.csv (for GSM2396859) on github.com/asncd/MIMOSCA. We did not consider the High MOI dataset due to the expected high number of duplicate sgRNA assignments. Cells were quality filtered based on expression, keeping the upper half of cells for each dataset. Only sgRNAs detected in at least 30 cells were used. For the day 7 dataset, 16,507, and for day 13 dataset, 9634 cells remained for benchmarking.

Ryan et al. measured knockdown effects of 50 transcription factors implicated in human definitive endoderm differentiation using a CRISPRi variant of CROPseq in human embryonic stem cells 6 days after transduction [21]. We obtained data of both replicates from GEO (GSM3630200, GSM3630201), which include sgRNA counts next to the rest of the transcription. We refrained from using the targeted sequencing of the sgRNA in GSM3630202, GSM3630203 as it contained less clear mappings due to amplification noise. Expression data lacked information on mitochondrial genes, and therefore, no further quality filtering of cells was performed. From this dataset, only sgRNAs detected in at least 100 cells were used. A combined 5282 cells remained for benchmarking.

Analysis was limited to the 10,000 most expressed genes for all three datasets.

We normalized the count matrices for each individual dataset (Perturb-Seq (7d), Perturb-Seq (13d), and CRISPRi) separately by using the R package *scran* (version 1.11.27) [42].

#### Human Cell Atlas study

This scRNA-seq dataset originates from a benchmark study of the Human Cell Atlas project and is available on GEO (GSE133549) [22]. The dataset consists of PBMCs and a HEK293T sample which was analyzed with 13 different scRNA-seq technologies (CEL-Seq2, MARS-Seq, Quartz-Seq2, gmcSCRB-Seq, ddSEQ, ICELL8, C1HT-Small, C1HT-Medium, Chromium, Chromium(sn), Drop-seq, inDrop). Most cells are annotated with a specific cell type/cell line (CD4 T cells, CD8 T cells, NK cells, B cells, CD14+ monocytes, FCGR3A+ monocytes, dendritic cells, megakaryocytes, HEK cells). Megakaryocytes (due to their low abundance) and cells without annotation were discarded from this analysis.

We normalized the count matrices for each technology separately by using the R package *scran* (version 1.11.27) [42].

### Dimensionality reduction with UMAP and assessment of cluster purity

We used the R package *umap* (version 0.2.0.0) calling the Python implementation of Uniform Manifold Approximation and Projection (UMAP) with the argument “method = ‘umap-learn’” to perform dimensionality reduction on various input matrices (gene expression matrix, pathway/TF activity matrix, etc.). We assume that the dimensionality reduction will result in clustering of cells that corresponds well to the cell type/cell type family. To assess the validity of this assumption, we assigned a cell-type/cell family-specific cluster-id to each point in the low-dimensional space. We then defined a global cluster purity measure based on silhouette widths [47], which is a well-known clustering quality measure.

Given the cluster assignments, in the low-dimensional space, for each cell, the average distance (*a*) to the cells that belong to the same cluster is calculated. Then, the smallest average distance (*b*) to all cells belonging to the newest foreign cluster is calculated. The difference, between the latter and the former, indicates the width of the silhouette for that cell, i.e., how well the cell is embedded in the assigned cluster. To make the silhouette widths comparable, they are normalized by dividing the difference with the larger of the two average distances $s=\frac{b-a}{\max \left(a,b\right)}$. Therefore, the possible values for the silhouette widths lie in the range − 1 to 1, where higher values indicate good cluster assignment, while lower values close to 0 indicate poor cluster assignment. Finally, the average silhouette width for every cluster is calculated, and averages are aggregated to obtain a measure of the global purity of clusters. For the silhouette analysis, we used the R package *cluster* (version 2.0.8).

For statistical analysis of cluster quality, we fitted a linear model *score = f(scRNA-seq protocol + input matrix)*, where *score* corresponds to average silhouette width for a given scRNA-seq *protocol* - *input matrix* pair. *Protocol* and *input matrix* are factors, with reference level Quartz-Seq2 and positive control, respectively. We fitted two separate linear models for transcription factor and pathway activity inference methods. We report the estimates and *p* values for the different coefficients of these linear models. Based on these linear models, we performed a two-way ANOVA and pairwise comparisons using TukeyHSD post hoc test.

### Comparison of PBMCs TF activity with gene essentiality

For each scRNA-seq technology and used TF analysis tool, we calculated mean TF expression for each PBMC type. To focus solely on PBMCs, cells classified as HEK cells or unknown were discarded from this analysis. In addition, we removed megakaryocytes because their abundance was in general too low across all technologies. We used the DepMap shRNA screen [31] as gene essentiality data. As a given TF can either increase proliferation (oncogene) or decrease it (tumor suppressor), we can expect either negative or positive correlation (respectively) between gene essentiality and TF activity. To correct for this effect, we calculated Pearson correlations between TF expression (from CCLE data [48]) and TF essentiality for each TF and multiplied TF essentiality values by the sign of this correlation coefficients. For categorizing hematologic cancers into myeloid and lymphoid groups, we used CCLE metadata (Additional file 4). Basically, we classified myeloid leukemias as myeloid and lymphoid leukemias and lymphomas as lymphoid cancers. Ambiguous cancer types were removed from our analysis.

## Supplementary information

**Additional file 1.** Supplementary figures S1-S15.

**Additional file 2.** Metadata of bulk RNA-seq TF perturbation data, including among others perturbation target, perturbation direction, GEO accession ID and annotated GEO sample IDs (whether samples belong to control or perturbation group). Those data were used to simulate single cells. (CSV 13 kb)

**Additional file 3.** Metadata of bulk RNA-seq pathway perturbation data, including among others perturbation target, perturbation direction, GEO accession ID and annotated GEO sample IDs (whether samples belong to control or perturbation group). Those data were used to simulate single cells. (CSV 1 kb)

**Additional file 4.** Manual classification of selected hematologic cancer cell lines from the CCLE database into myeloid (M) or lymphoid (L) cancer. (CSV 2 kb)

**Additional file 5.** Review history.

## Data Availability

The code to perform all presented studies is written in R [49, 50] and is freely available on GitHub: https://github.com/saezlab/FootprintMethods_on_scRNAseq [51]. The datasets supporting the conclusions of this article are available at Zenodo: 10.5281/zenodo.3564179 [52].
