# Supplementary material for: Robustness and applicability of transcription factor and pathway analysis tools on single-cell RNA-seq data
Source: Genome Biol. 2020 Feb 12;21:36. doi: 10.1186/s13059-020-1949-z (PMC7017576; doi:10.1186/s13059-020-1949-z)
Supplement: Supplementary file 1 — Additional file 1. Supplementary figures S1-S15. [file 13059_2020_1949_MOESM1_ESM.docx]

**Supplementary Figures for**

**“Robustness and applicability of transcription factor and pathway analysis tools on single-cell RNA-seq data”**


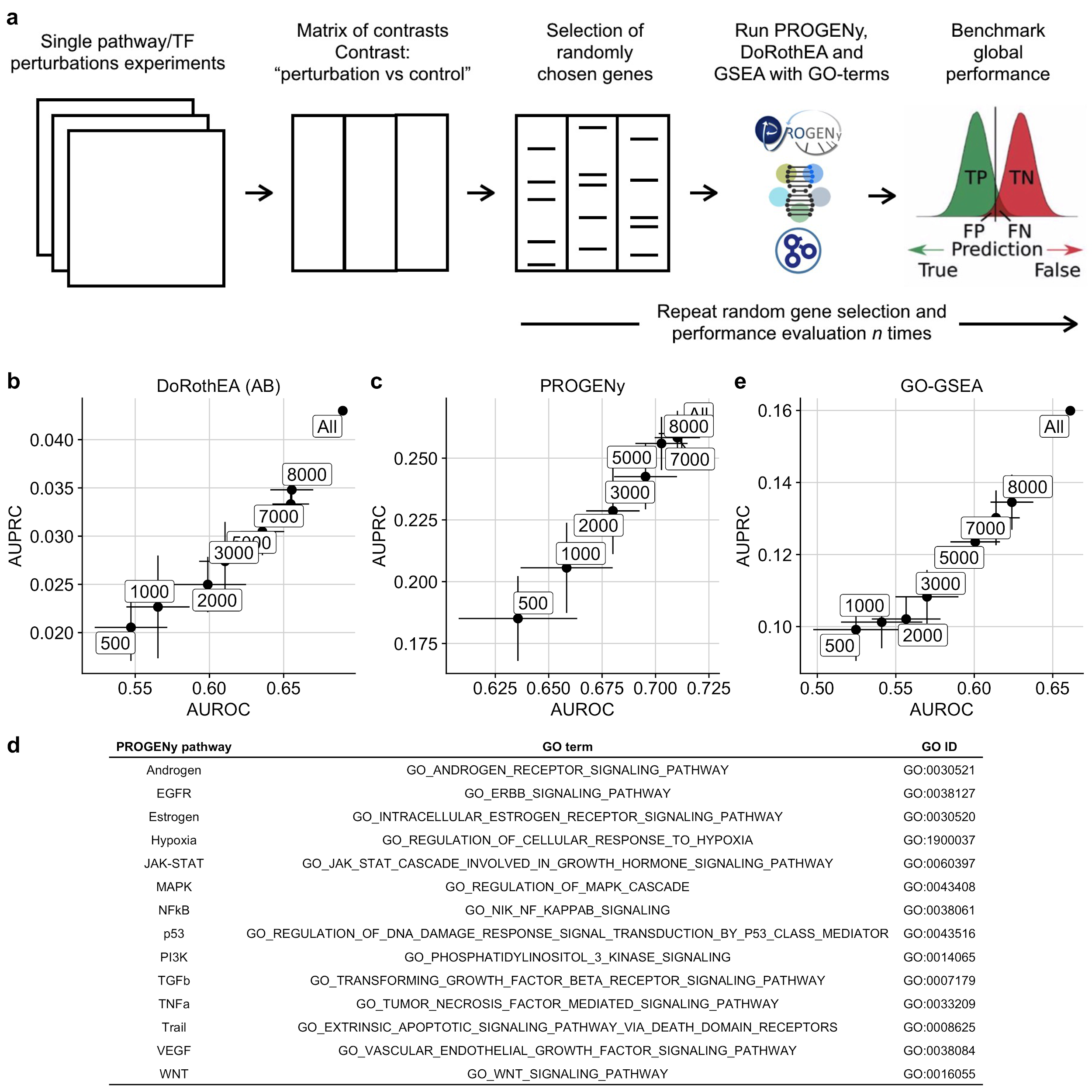
*Fig. S1:* ***a*** *Workflow to induce low gene coverage with a subsequent benchmark of the tools PROGENy, DoRothEA and GO-GSEA against low gene coverage.* ***b,c,e*** *Scatterplot showing how well AUROC and AUPRC of* ***b*** *DoRothEA (AB),* ***c*** *PROGENy with 100 footprint genes per pathway, and GO-GSEA are correlated. The labels correspond to the gene coverages.* ***d*** *Mapping table between PROGENy pathways and GO terms/GO IDs.*


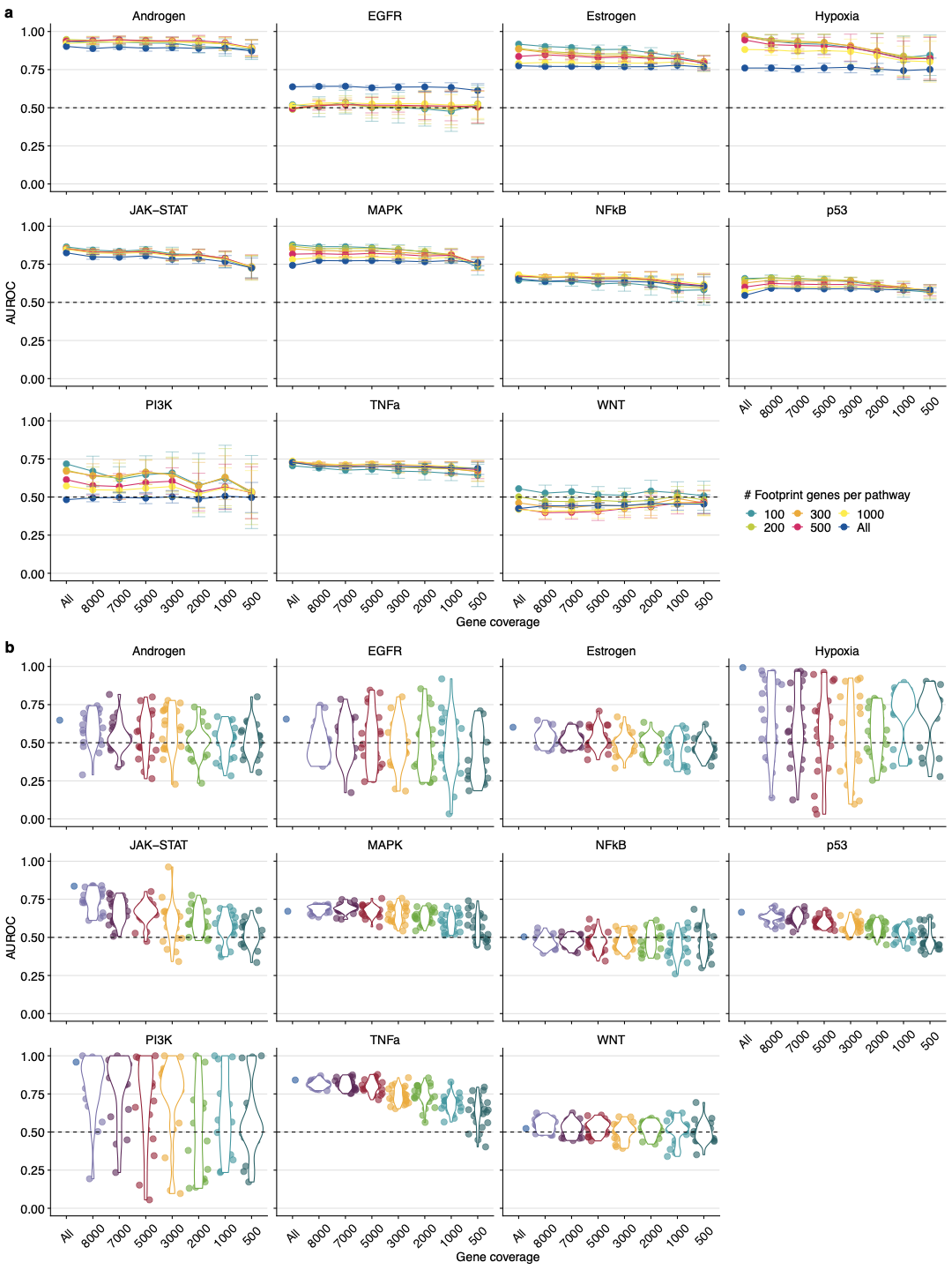


*Fig. S2: Pathway-wise evaluation of* ***a*** *PROGENy and* ***b*** *GO-GSEA at different gene coverages. Performance is measured as Area under the ROC curve (AUROC). The dashed line indicates the performance of a random model. The colors in* ***b*** *are meant only as a visual support to distinguish between the individual violin plots and jittered points.*


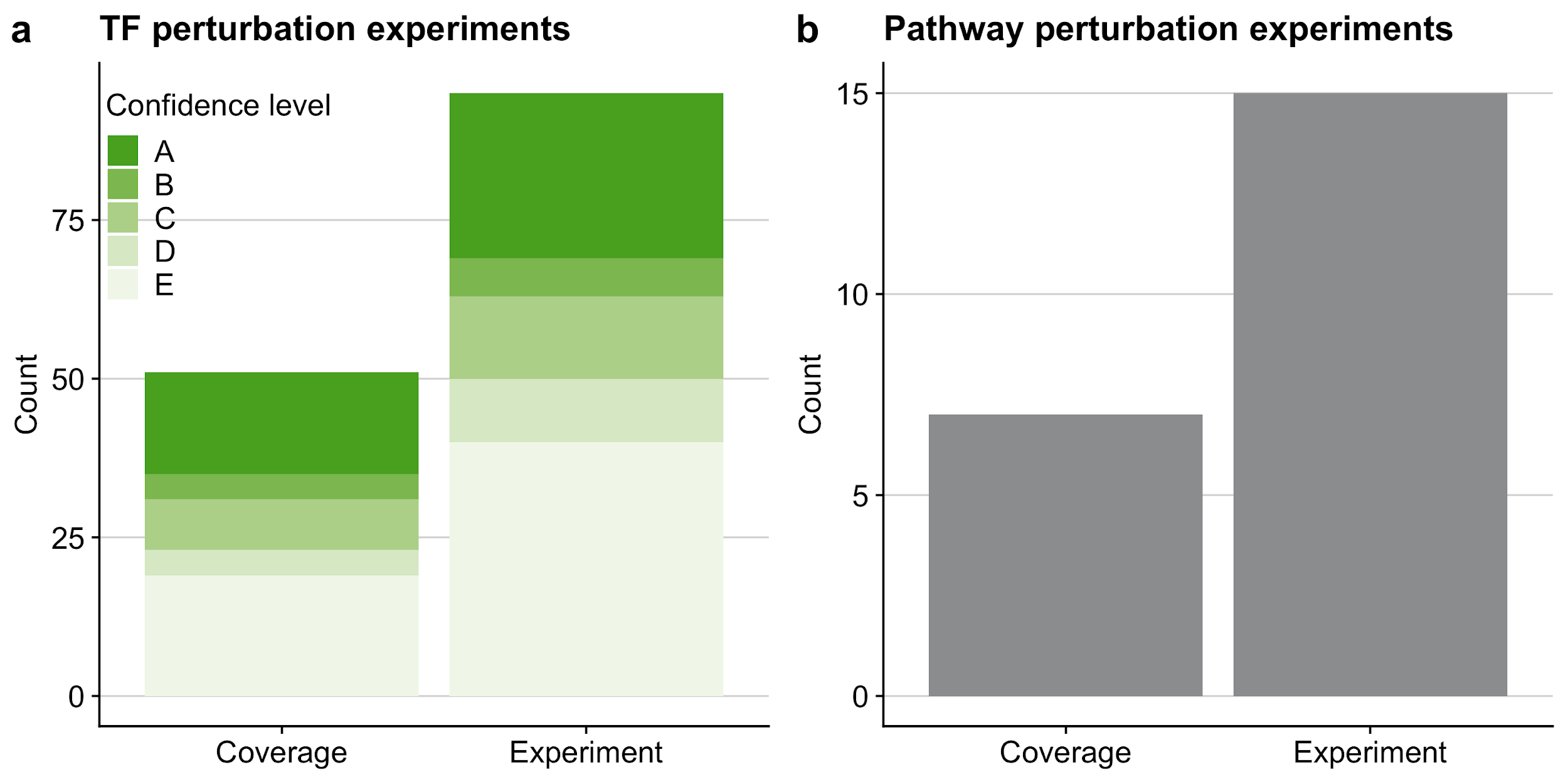


*Fig. S3: Overview of the benchmark dataset of the in silico study for* ***a*** *TF and* ***b*** *pathway analysis tools. The term coverage denotes the number of distinct perturbed TFs and pathways in the benchmark dataset covered by the respective gene set resource. As individual pathways/TFs can be perturbed several times in independent experiments we also provide the total number of perturbation experiments. In the case of TF perturbation experiments we also provide DoRothEA’s confidence class for each perturbed TF indicating the quality of its regulon within DoRothEA (A - high quality to E - low quality).*


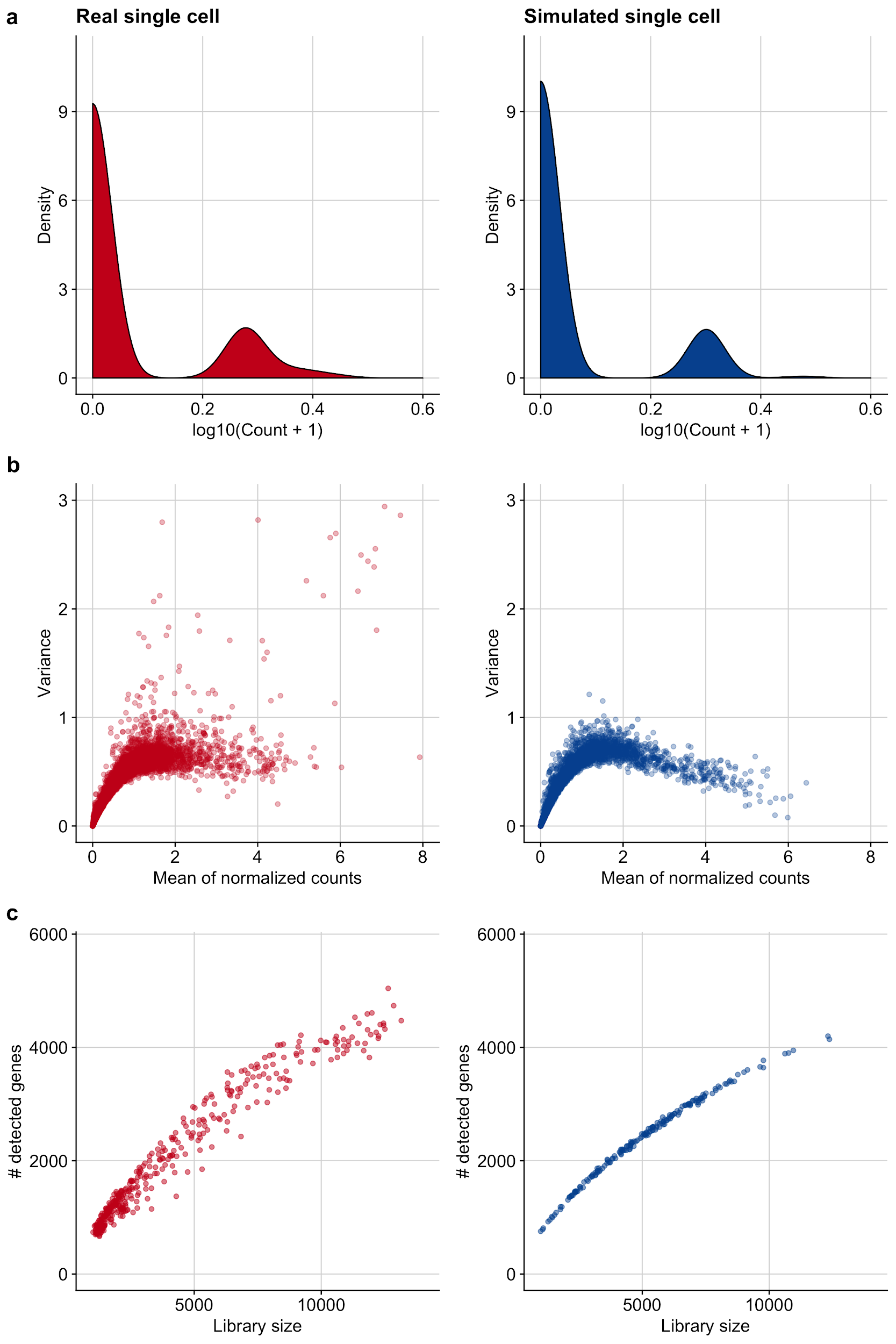
*Fig. S4: Comparison of single-cell-specific properties between real and simulated single cells.* ***a*** *Count distribution of a representative gene for a real and a simulated single cell.* ***b*** *Mean-variance relationship of gene expression of a representative data set for a real and a simulated single cell.* ***c*** *The dependence of the number of detected genes in a real and a simulated single cell on the library size.*


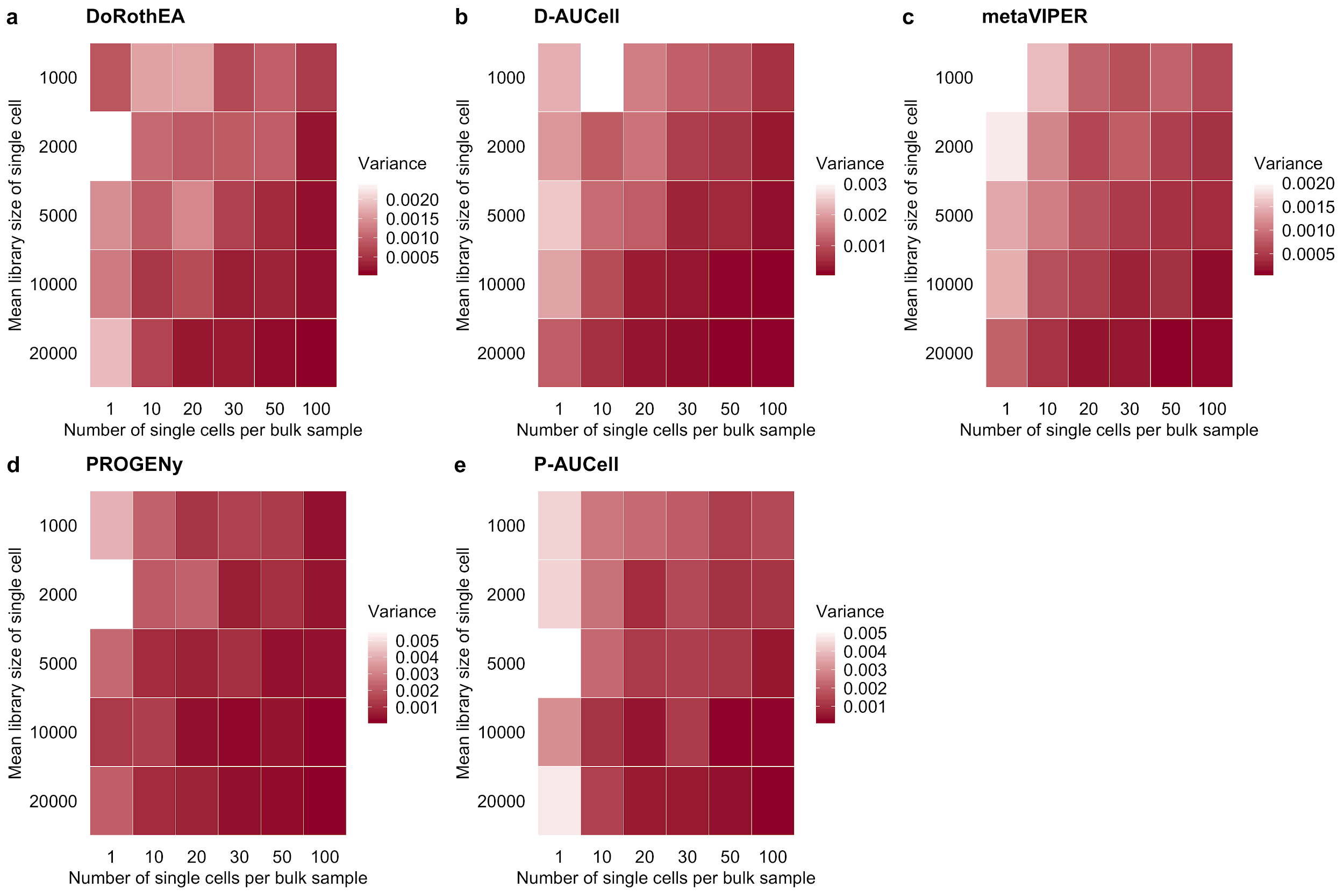
*Fig. S5: Variance in the performance (measured as AUROC) of* ***a*** *DoRothEA,* ***b*** *D-AUCell,* ***c*** *metaVIPER,* ***d*** *PROGENy and* ***e*** *P-AUCell on single cells for different combinations of simulation parameters. The variance is calculated by repeating the simulation of each single-cell for each parameter combination 25 times.*


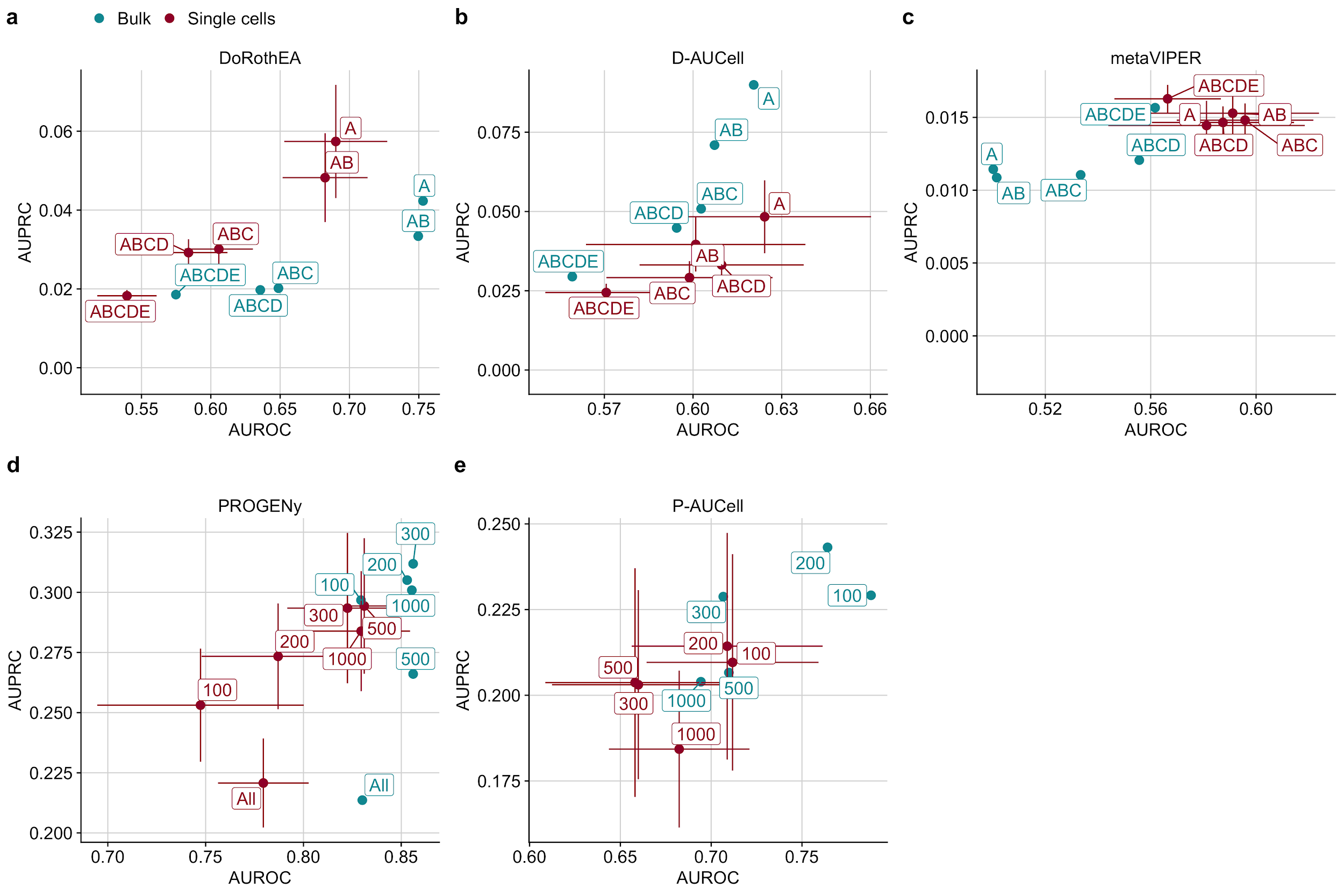


*Fig. S6: Scatterplot comparing the performance of* ***a*** *DoRothEA,* ***b*** *D-AUCell,* ***c*** *metaVIPER,* ***d*** *PROGENy, and* ***e*** *P-AUCell on single cells and bulk, measured with AUROC and AUPRC with respect to different combinations of* ***a,b,c*** *DoRothEA’s confidence levels or* ***d,e*** *different number of footprint genes per pathway.*


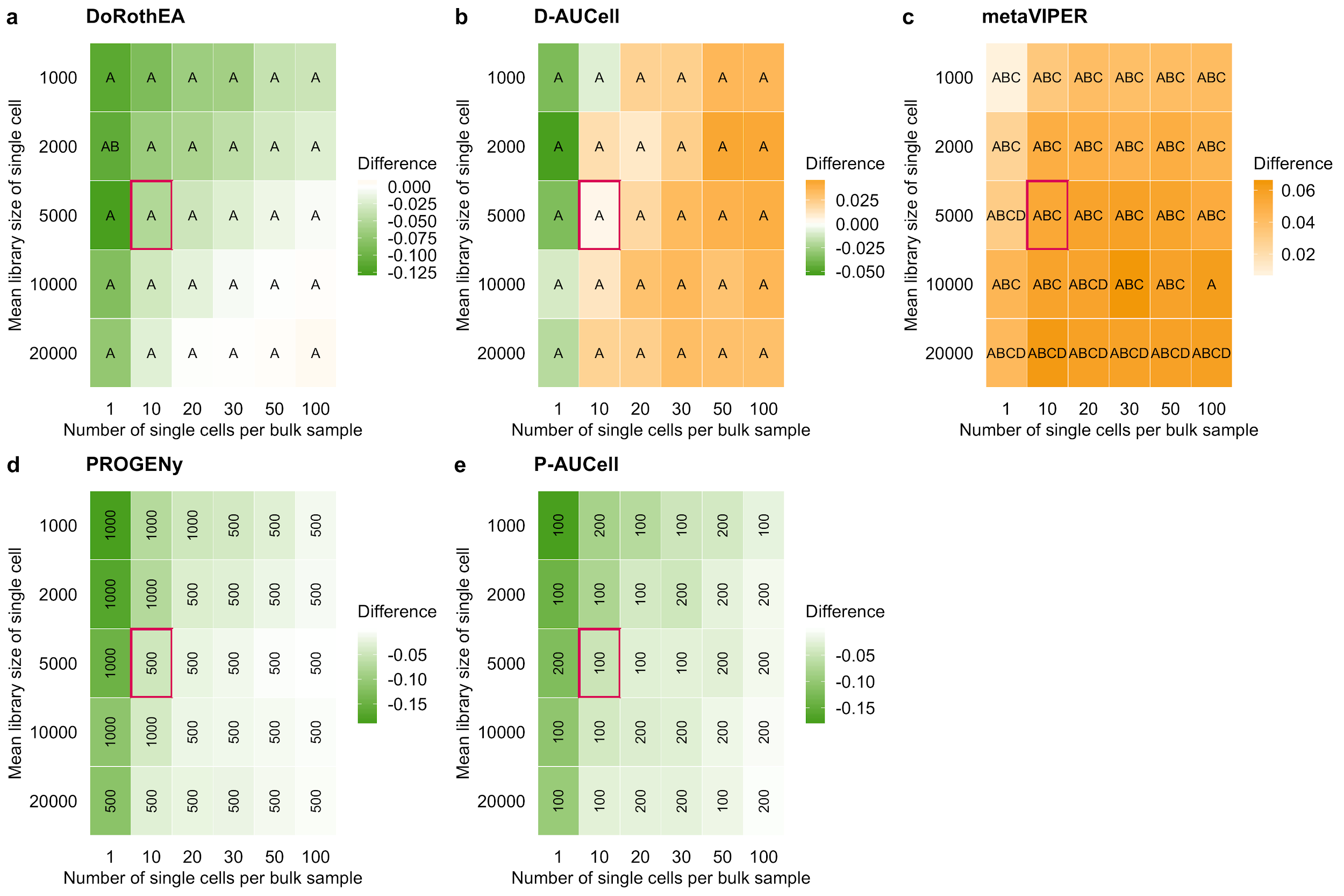


*Fig. S7: Effect of the simulation parameters on the performance of TF and pathway analysis tools. The tile plots show the difference in performance of* ***a*** *DoRothEA,* ***b*** *D-AUCell,* ***c*** *metaVIPER,* ***d*** *PROGENy, and* ***e*** *P-AUCell between single cells and corresponding bulk samples,* ***a,b,c*** *across all confidence level combinations or* ***d,e*** *different number of footprint genes per pathway. A negative value indicates that the performance on bulk was better than on the simulated single cells and vice versa. The letters/numbers within the tiles indicates which confidence level combination/number of footprint genes per pathway performed the best on the single-cell data for the given parameter combination. The tile marked in red corresponds to the parameter setting used for previous plots (Fig. 2)*


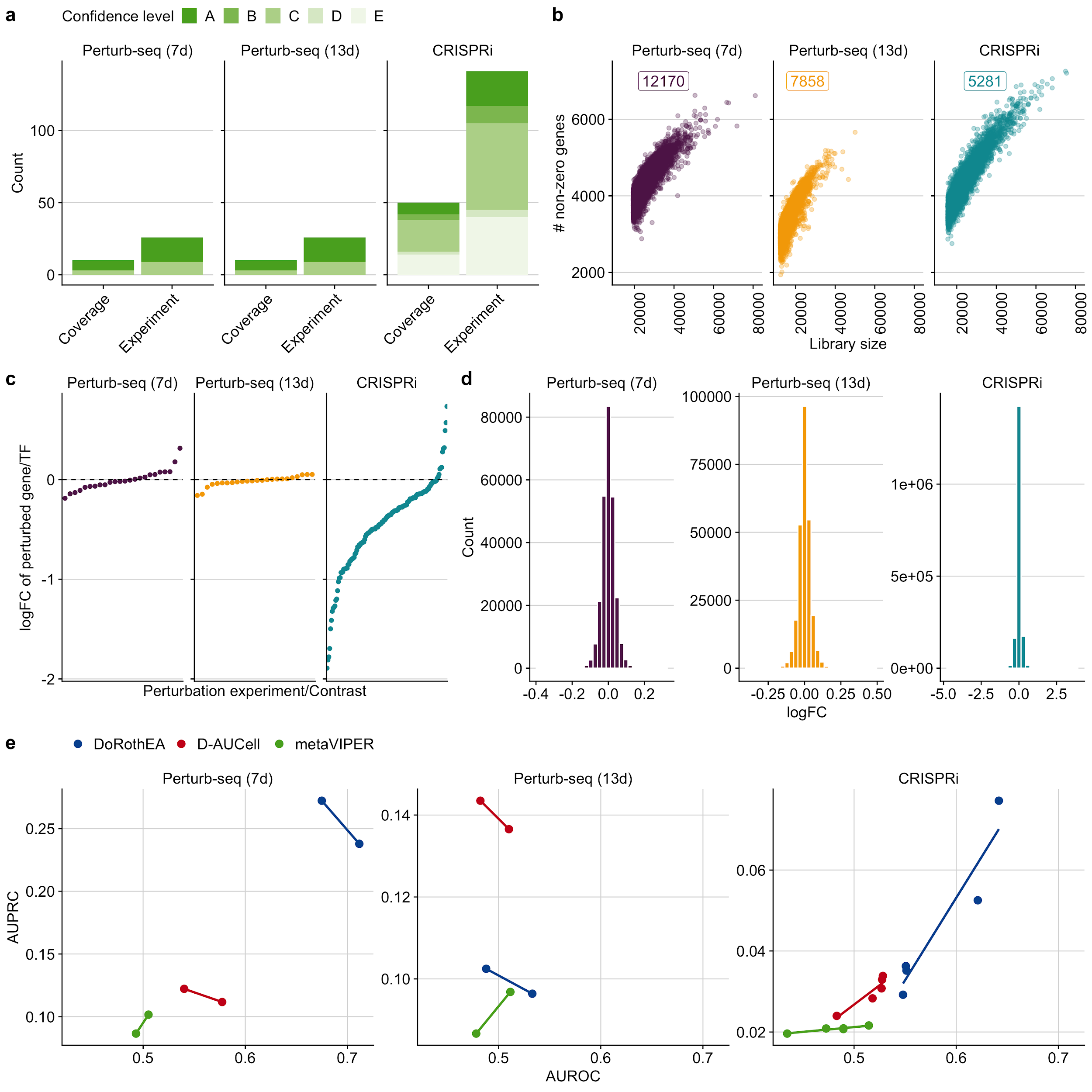
*Fig. S8:* ***a*** *Overview of the in-vitro benchmark dataset. The term coverage denotes the number of distinct perturbed TFs in the benchmark datasets. As individual TFs can be perturbed several times in independent experiments we also provide the total number of perturbation experiments. We also provide DoRothEA’s confidence class for each TF indicating the quality of its regulon (A - high quality to E - low quality).* ***b*** *The dependence of the number of detected genes on the library size for all benchmark datasets. The number of corresponding cells are displayed as well.* ***c*** *logFC of perturbed target/TF for the corresponding perturbation experiment for all benchmark datasets.* ***d*** *Distribution of logFC of all genes for each benchmark dataset.* ***e*** *Relationship between AUROC and AUPRC for DoRothEA, D-AUCell and metaVIPER with respect to different combinations of DoRothEA’s confidence levels for each benchmark dataset.*


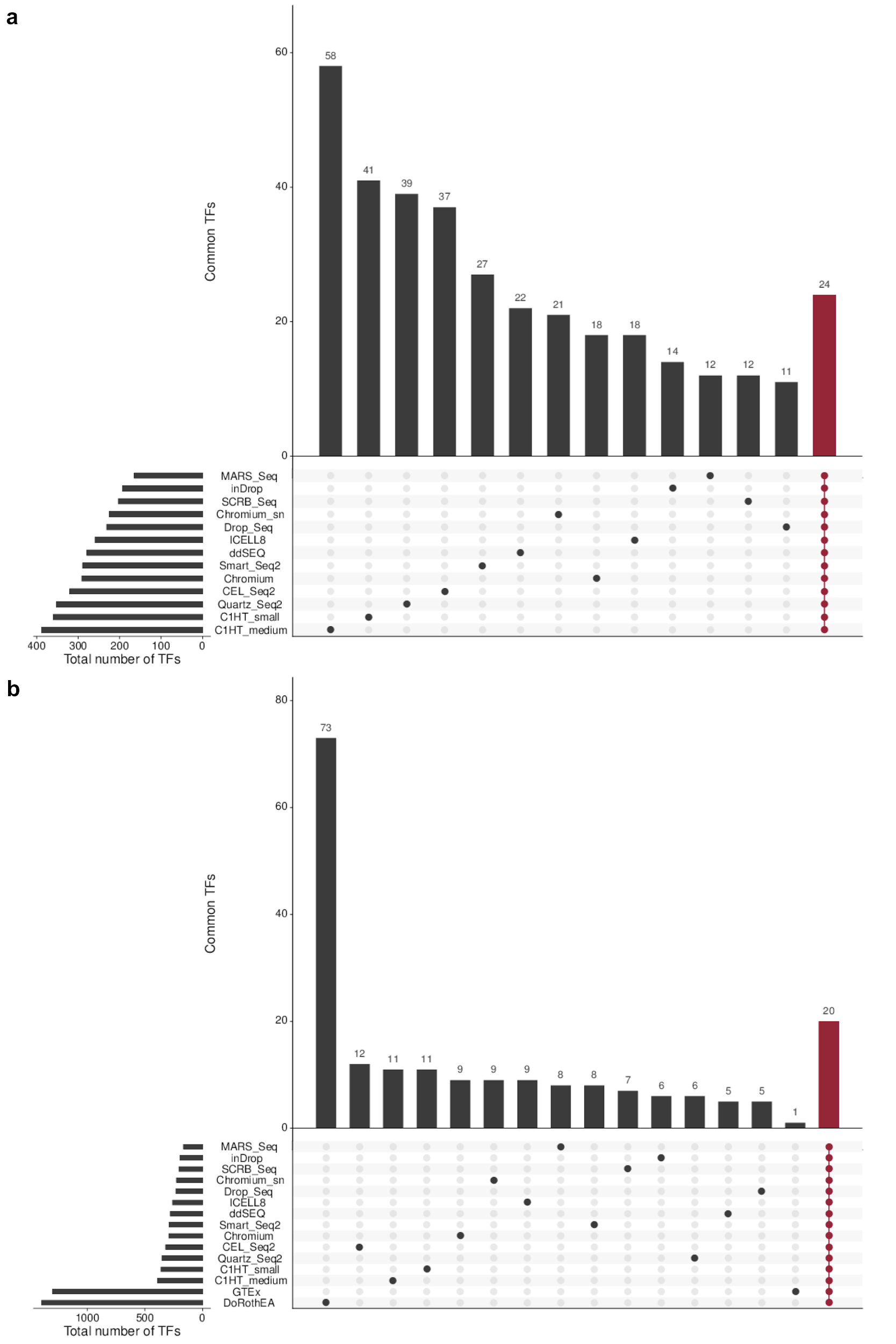
*Fig. S9: Overlap of TF regulon resources.* ***a*** *Overlapping TFs of protocol-specific SCENIC regulatory networks. All 13 networks share 24 TFs.* ***b*** *Overlapping TFs between protocol-specific SCENIC regulatory networks, GTEx regulons and DoRothEA. All resources share 20 TFs. The remaining vertical bar plots indicate the number of TFs that are exclusive for the respective regulon resource. The horizontal bar plots indicate the total number of TFs for the regulon resource.*

*
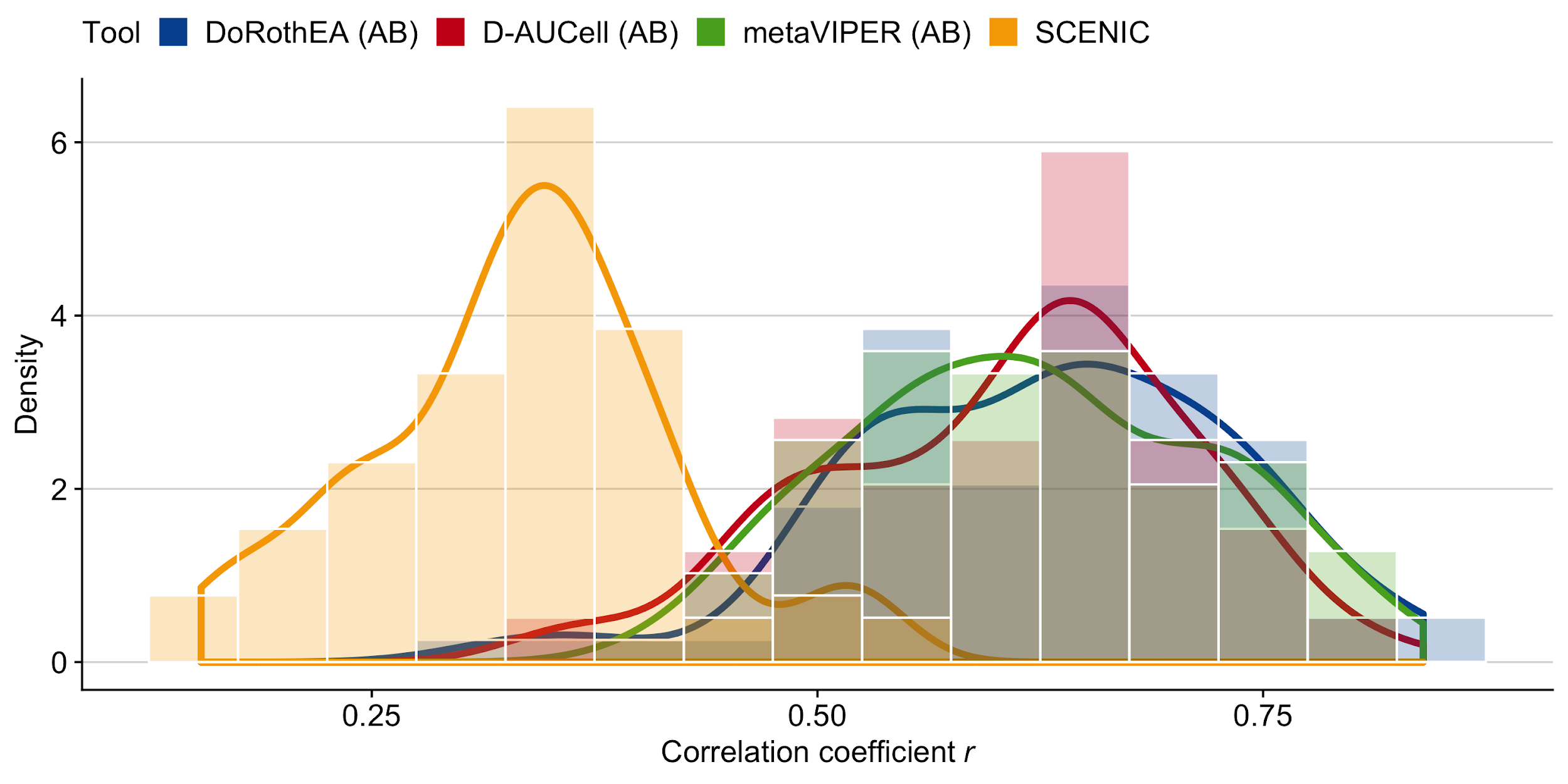
*

*Fig. S10: Pairwise (Pearson) correlations of TF activities between the scRNA-technologies for each TF analysis tool.*


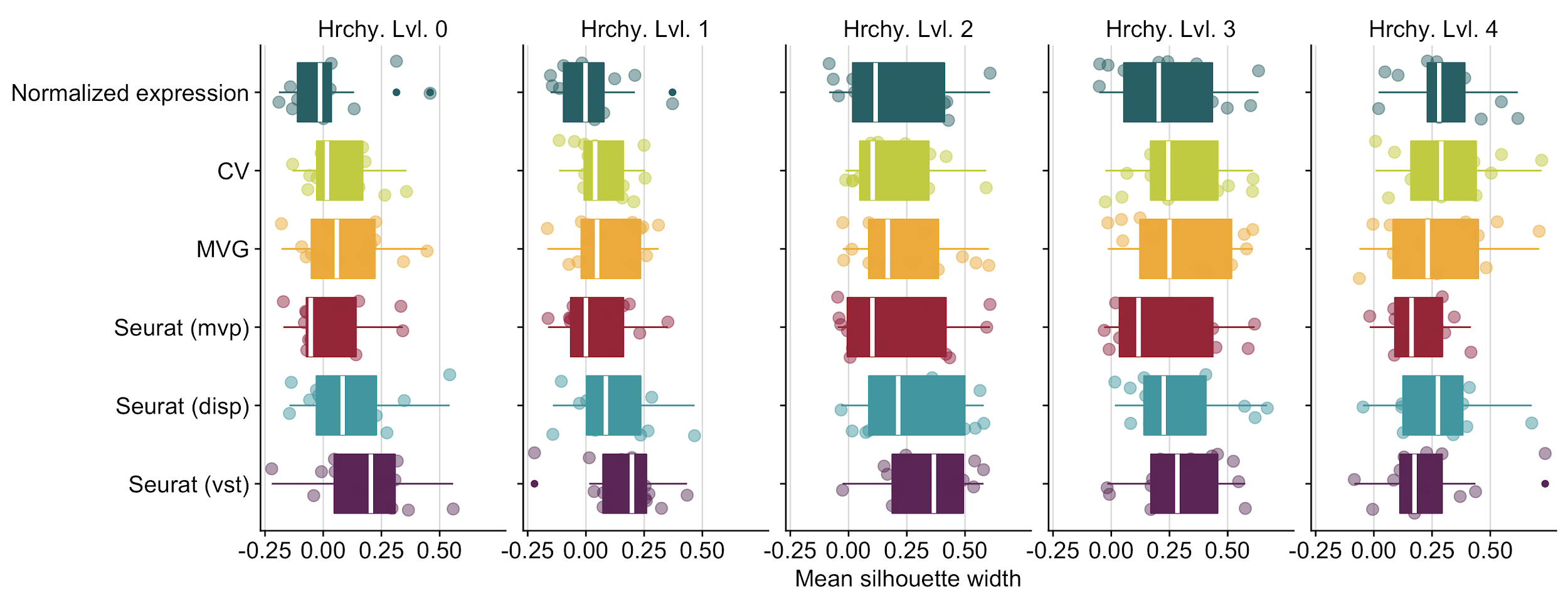
*Fig. S11. Identification of the best method to determine the top 2000 highly variable genes to be considered for dimensionality reduction. We tested three different selection methods implemented in Seurat (disp = dispersion, mvp = mean.var.plot, vst). We also included CV (squared coefficient of variation - (sd/mean)**2) and MVG (most variable genes - genes with the highest variance). Those methods are compared to the case of considering the full gene expression matrix for dimensionality reduction, indicated here as ‘Normalized expression’.*


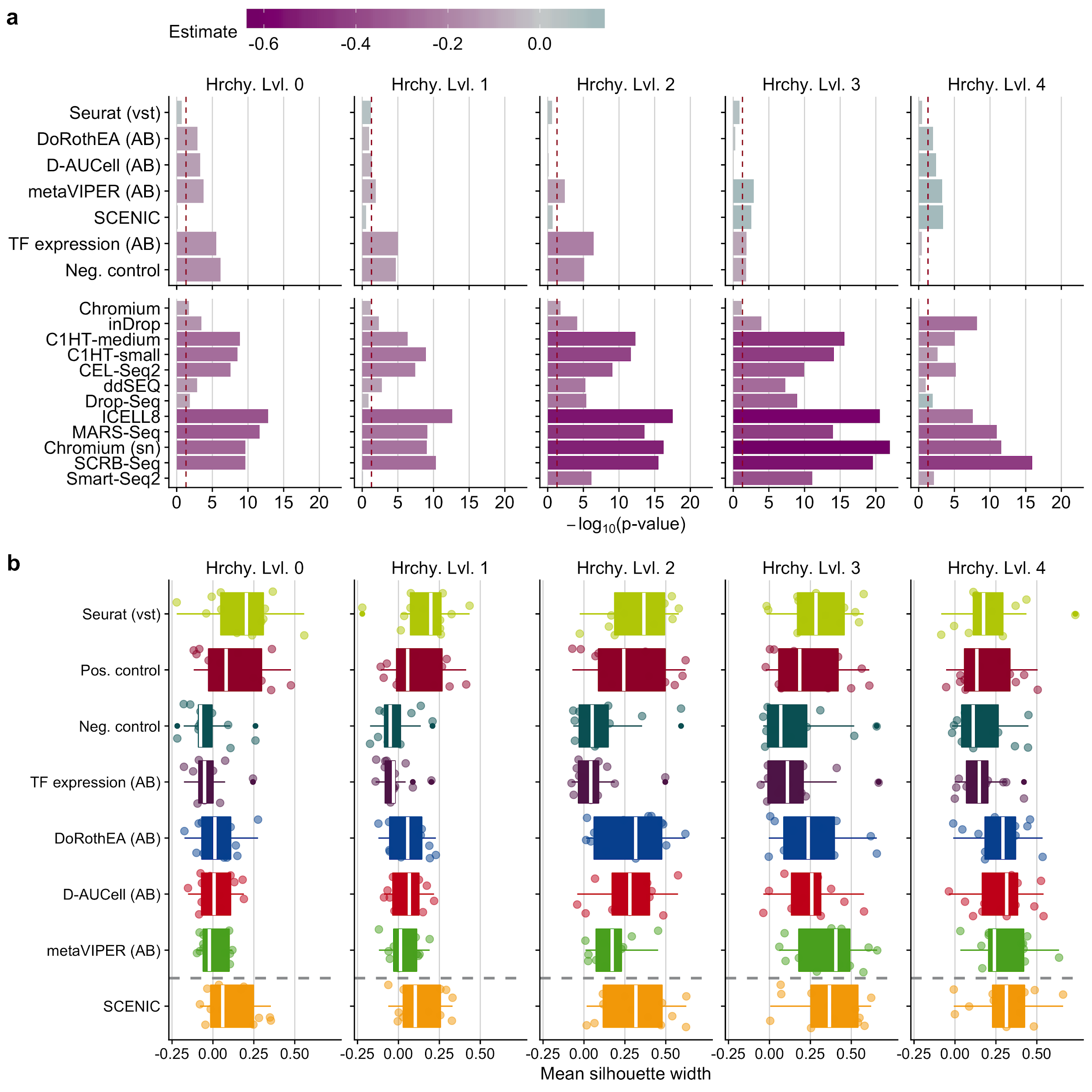
*Fig. S12:* ***a*** *Statistical analysis of cell cluster purity in the reduced space: i) differences in the quality of clustering with respect to the positive control and ii) quality of clustering of scRNA-seq protocols in contrast to Quartz-Seq2 for TF activity tools. This analysis was performed independently for all hierarchy levels (Hrchy. Lvl.). The legend key ‘estimate’ corresponds to the estimated coefficients of the linear model. A negative value indicates a worse performance than the reference level (positive control for input matrices and Quartz-Seq2 for protocols) and vice versa.The dashed line indicates a p-value of 0.05.* ***b*** *Comparison of cluster purity measured by the silhouette widths obtained when considering highly variable genes identified by Seurat, TF analysis tools and controls for all hierarchy levels.*


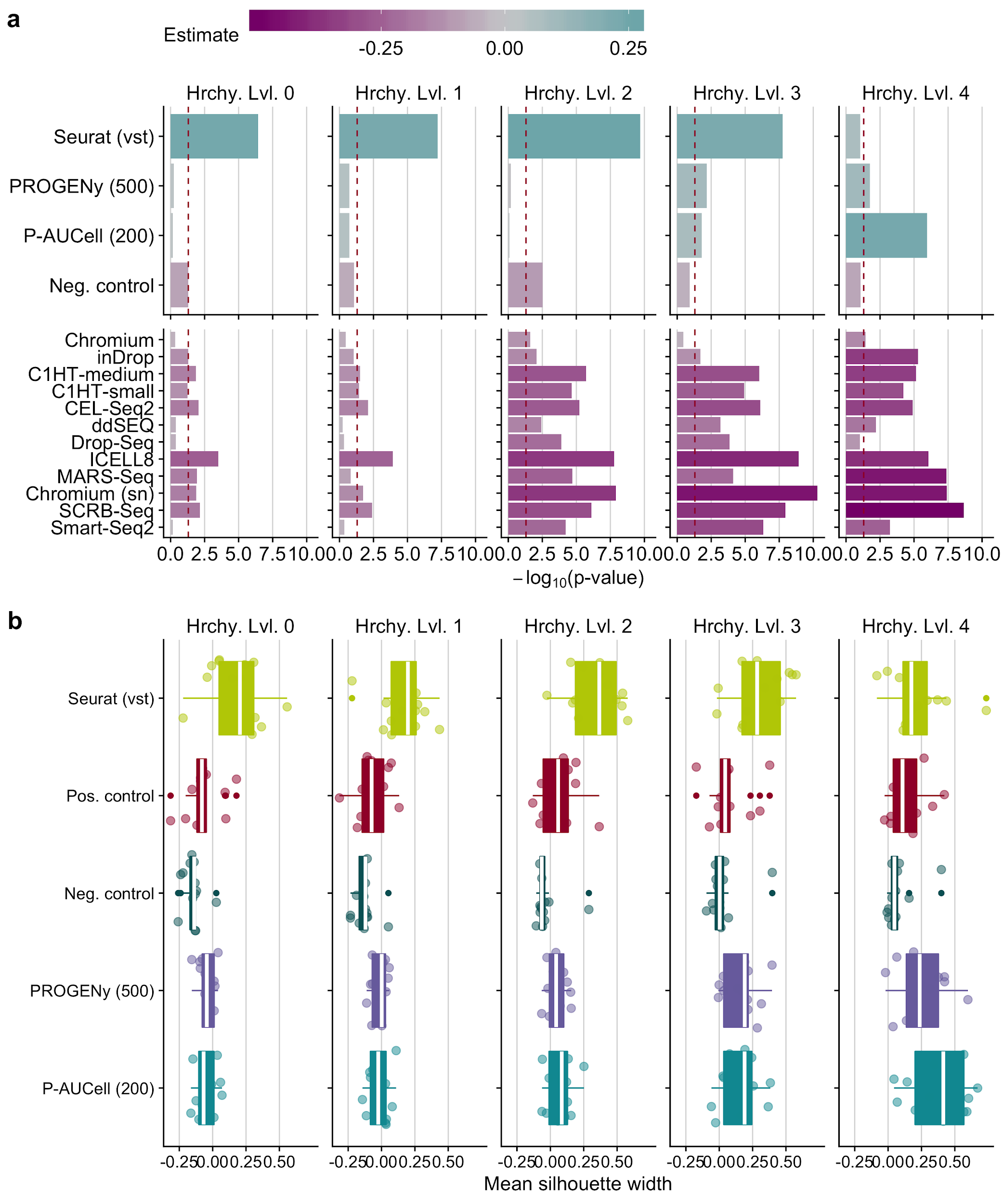
*Fig. S13:* ***a*** *Statistical analysis of cell cluster purity in the reduced space : i) differences in the quality of clustering using different input matrices with respect to positive control and ii) quality of clustering of scRNA-seq protocol in contrast to Quartz-Seq2 for pathway activity inference tools. This analysis was performed independently for all hierarchy levels (Hrchy. Lvl.). The legend key ‘estimate’ corresponds to the estimated coefficients of the linear model. A negative value indicates a worse performance than the reference level (positive control for input matrices and Quartz-Seq2 for protocols) and vice versa. The dashed line indicates a p-value of 0.05.* ***b*** *Comparison of cluster purity measured by the silhouette widths obtained when considering highly variable genes defined by Seurat, pathway analysis tools and controls for all hierarchy levels.*


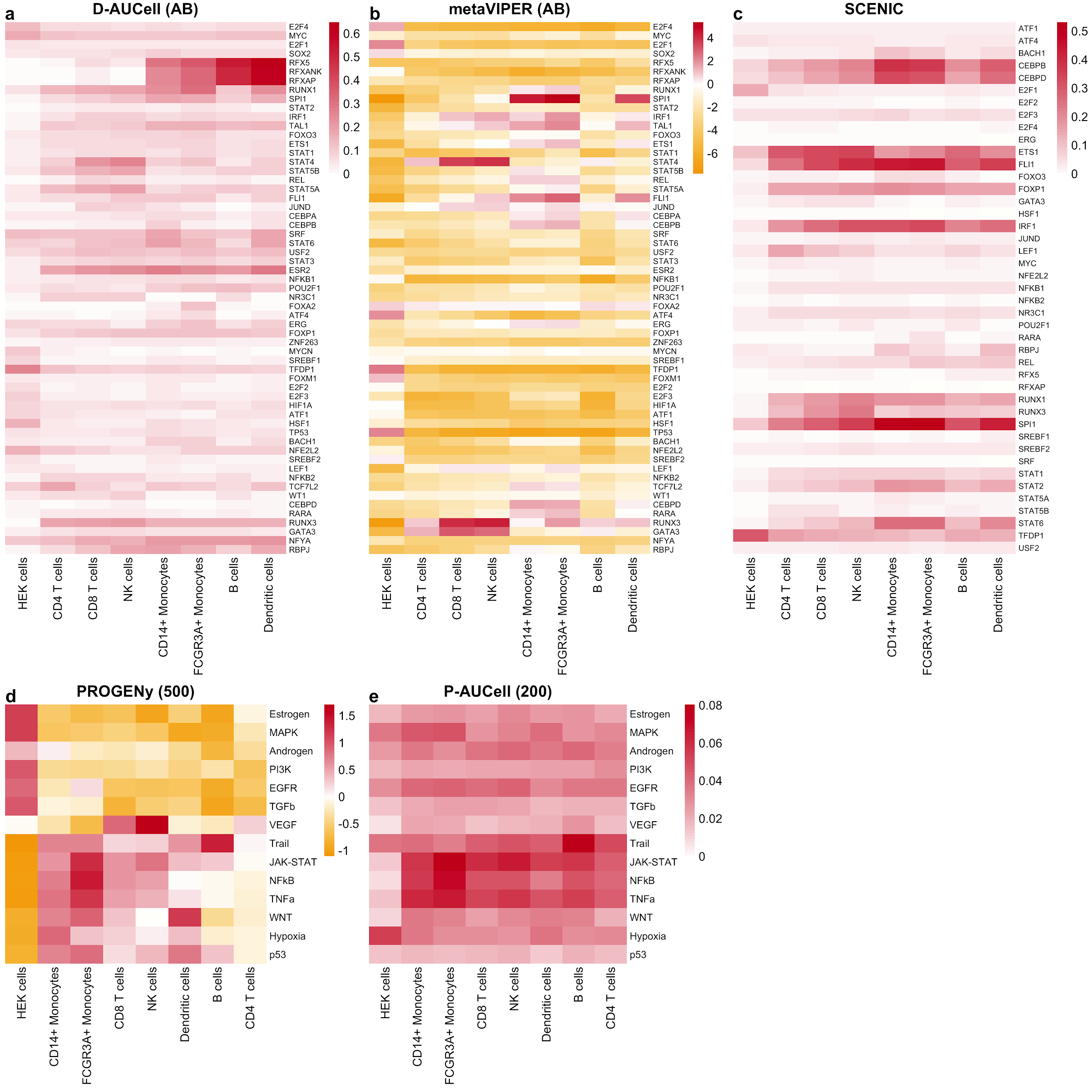
*Fig. S14: Selected TF and pathway activities of various tools inferred from the Quartz-Seq2 gene expression data summarized for each cell type/cell line separately. The letters in the brackets correspond to DoRothEA’s confidence levels and the numbers in brackets correspond to the number of footprint genes per pathway.*


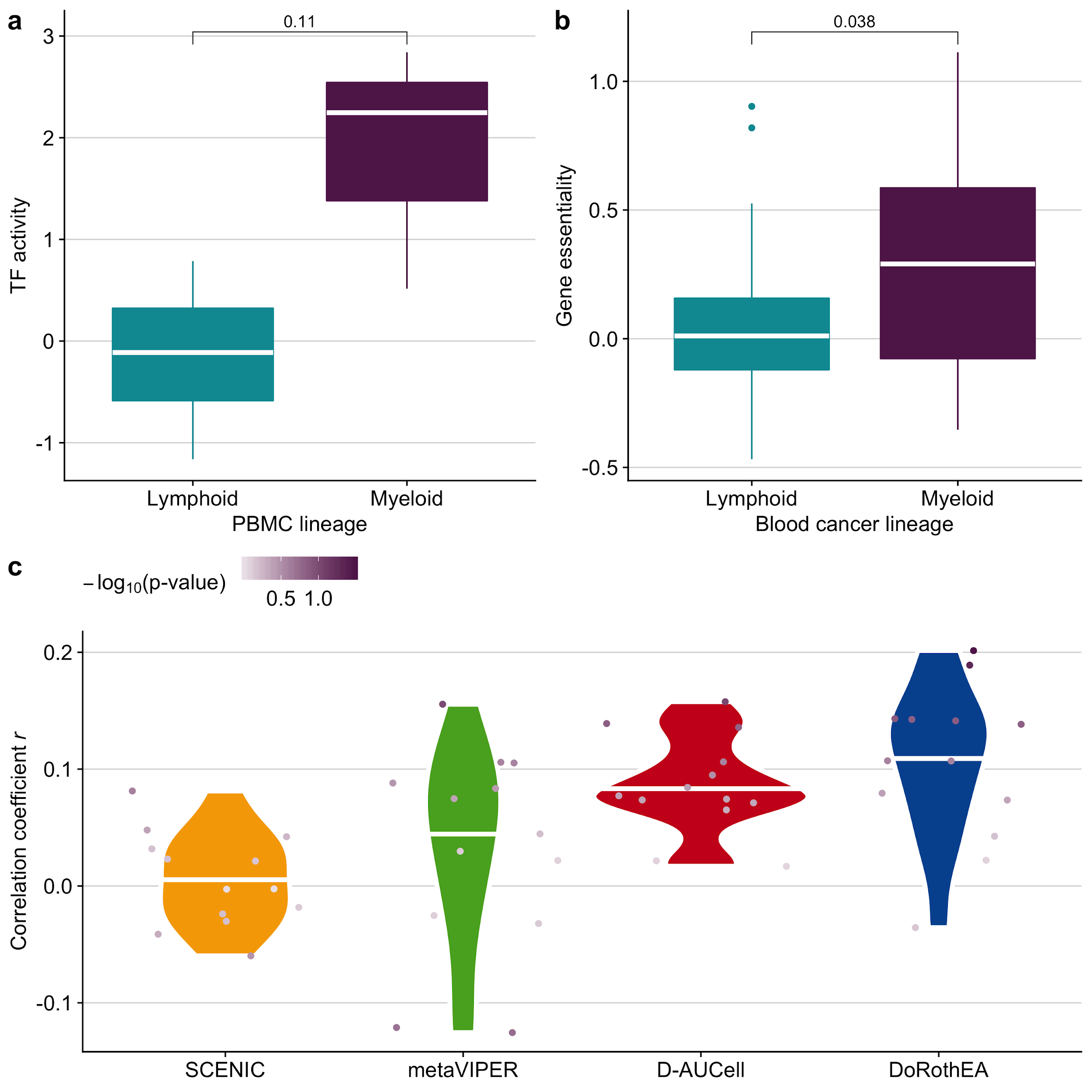


*Fig. S15:* ***a*** *TF activity and* ***b*** *gene essentiality for the TF/gene SPI1 in lymphoid and myeloid cells.* ***c*** *Correlation of the differences in TF activity and differences in gene essentiality separately for all tool-protocol combinations. The results are summarized for each TF analysis tool. The points on the violin plots represent the different scRNA-seq protocols.*
