# Supplementary material for: Robustness and applicability of transcription factor and pathway analysis tools on single-cell RNA-seq data
Source: Genome Biol. 2020 Feb 12;21:36. doi: 10.1186/s13059-020-1949-z (PMC7017576; doi:10.1186/s13059-020-1949-z)
Supplement: Supplementary file 5 — Additional file 5. Review history. [file 13059_2020_1949_MOESM5_ESM.docx]

Review History

**First round of review**

**Reviewer 1**

**Are you able to assess all statistics in the manuscript, including the appropriateness of statistical tests used?**

Yes, and I have assessed the statistics in my report.

**Comments to author:**

Holland et al. presents comparisons of multiple approaches to predict active pathways and TF activities from single-cell RNA-seq data. Specifically, they evaluated the capacity of PROGENy and DoRothEA gene sets combined with the statistics implemented in the AUCell and VIPER tools to retrieve TF and pathway activities on in silico and in vitro sc-RNAseq data. Moreover, they assessed the capacity of these approaches to cluster distinct cell types on 13 scRNA-seq datasets (different protocols on the same cell types). The authors claim that tools developed for bulk functional genomics can be applied to scRNA-seq data, outperforming single cell tools. The study is of high interest to the community, the manuscript well written, and the analyses overall well performed.
However, the analyses do not fully support the main claim of the paper, which should be at least soften. Specific comments are provided hereafter.

1. The main claim of the authors right from the abstract is that "bulk functional genomics tools can be applied to scRNA-seq data, outperforming dedicated single cell tools." However, their clearly state in the discussion that their analyses suggest that "the performance of functional genomics tools is more sensitive to the selection of gene sets than the statistical methods." Hence, the comparisons performed by the authors might be biased due to the specific sets of genes they used (namely from DoRotheEA and PROGENy). This is confirmed by the fact that metaVIPER is performing poorly while VIPER on DoRothEA performs better. Hence, AUCell might perform better than VIPER on another gene set than DoRothEA.

2. The authors claim that "high confidence TF regulons from DoRothEA outperform pure ARACNe regulons," emphasizing "the importance of combining literature curated resources with in silico predicted resources". It would be interesting to see how the SCENIC tool (relying on AUCell) performs on identifying key TFs involved in driving the different cell types and its capacity to cluster sc-RNAseq. Indeed, in its associated publication, SCENIC is described as able to identify TF regulons automatically (without relying on prior knowledge such as DoRotheEA) and use this information to cluster cells. The analyzed datasets and metrics used to assess the clustering capacity of SCENIC in its publication could be used for comparison with DoRothEA. As such, SCENIC goes one step beyond the combination of VIPER and DoRothEA as it predicts TF regulons and TF activities in single cells and uses it to cluster cell types.

3. The title would need to be more specific as the evaluation was specific to TF and pathway activities estimation and not all "functional genomic tools".

**Reviewer 2**

**Are you able to assess all statistics in the manuscript, including the appropriateness of statistical tests used?**

No, I do not feel adequately qualified to assess the statistics.

**Comments to author:**

Functional analysis of genomics data has greatly increased our understanding of our organs and tissues are specified and how they function. A large literature exists that describes the gene regulatory networks or pathway activity analysis from bulk tissue-specific RNA sequencing data.
Recently, there has been a revolution in technology that allows single cells to be profiled at the transcriptome level. Much as previously we have been able to understand the transcriptional difference between tissues, it is now becoming possible to examine the difference between individual cells. However, to really obtain insights from this single cell data a number of computational challenges need to be addressed. First and foremost is the sparsity of scRNAseq data. Due to our inability to capture all transcripts in a single cell, scRNAseq data has a very high drop-out rate where individual genes are not seen as being expressed primarily because of the stochasticity of message capture during the library preparation process. This has meant that different laboratories have started to develop algorithms that are specifically designed to analyse this scRNAseq data.

This manuscript compares whether these single cell specific algorithms perform better than the methods that have previously been developed for bulk data. This is a very timely and pertinent questions as the amount of scRNAseq data that is being generated is increasing at an astonishing pace.

The authors compare a number of algorithms that perform two distinct functions. Firstly algorithms that determine the activity of specific pathways, in particular the bulk analysis tools PROGENy and GO-enrichment are compared to AUCell,  and secondly algorithms that build gene regulatory networks (DoRothEA with VIPER for bulk) is compared to single cell GRN methods such as metaVIPER. For their analysis the authors use either bulk, simulated scRNAseq data and real scRNAseq data. Their analyses are appropriate for the questions asked.

Somewhat surprisingly to me, the authors find that the methods designed for bulk outperform those developed for single cells. For me also very interesting was the insight that the choice of gene sets used in these types of algorithms is more important than the choice of statistical method to assess enrichment of the particular gene set, and this could be highlighted in the abstract (Currently only mentioned in discussion).

Overall these conclusions are of interest to the rapidly growing single cell genomics community and deserve publication. However, in a number of areas I would have liked to see additional analysis or further information. I have made some suggestions below. As it stands I found the paper very hard to follow.

1.     The authors analyse TF perturbation data that was previously published. The results of the global analysis are analysed with ROC and precision recall curves. Whilst I am sure this is a mathematically robust way of presenting the analysis, my gut feeling is that this may not tell the whole story. For example, I would imagine that some pathways are very robust and invariably recalled, even when drop-out rates are high, whilst other pathways may be much more sensitive, for example because this pathway is not very active in the cell type analysed. Could some extra information be provided to give a sense of this ? ( If my assumption of this variability is correct) The spread of results is given (Fig 1) but I cannot tell if it is the same pathways that are always at the top of that range.
2.     Minor point: do the colours in Fig 1a and c mean anything?
3.     For their second analysis, (p8) the interest of the paper would be extended to a more generalist audience if this section briefly described and contrasted the key features of each of the methods in more detail.
4.     The next analysis is supposed to be illuminated by Fig s2. However, the figure legend and the labels are very uninformative. E.g. count: is this: counts per gene per cell? Coverage? Experiment? This requires much more detail to be interpretable. This also applies to a number of other figures.
5.     The analysis described in Fig 2 is not clearly explained. E.g on page 9: Parameter combination (number of cells= 10, mean library size=5000). I found the terms used not self-explanatory and ill-defined. Simulation of 10 cells seems to me to be very non-representative of any single cell data set (or are they referring to the number of times the simulation was carried out? ) Why was the number of 10 chosen for most of the analysis? Could the term mean library size be made clearer (total number of unique reads per cells or is it the total number of genes detected? )
6.     The term TF coverage is used without explaining what this actually is. I therefore do not understand the analysis presented in figure 2b, step2.
7.     Step 3: "absolute difference"- this is not explained in the results.
8.     In Fig 3 the most notable feature is that in two out of three panels the AUROC values were very low. Perturb-seq is a difficult experiment to carry out. Is this the most appropriate data set to test the performance of the algorithms on? Whilst the authors state that the ground truth is known in these experiments, I wonder whether this is really the case.
9.     The final analysis shows some very interesting trends, especially the good cluster separation when using TF activity rather than TF expression, although even TF activity performed less well than to top HVG (Fig 4b). Overall the figure highlights the value of directionality (ie activation or repression) within the algorithms that assesses the TF activity. Given that DoRothEA makes specific predictions about which TFs are likely to be essential for each of these cell types, can the authors validate any of their conclusions. Can they either generate this data or compare it to data that is out there? E.g TF essentiality data may be of use here; or TF dependencies in cancer data.  

In conclusion, I feel the authors have a very interesting story to tell. However, more background info on the analysis that are carried out would make this paper much more accessible. Figure legends and precise definitions of terms used could be greatly improved.  
 
Minor errors/typos:
page 5: Specifically, we (no capital)
pae 17; line 415- that should be than

**Authors Response**

**Point-by-point responses to the reviewers’ comments:**

Reviewer #1

Holland et al. presents comparisons of multiple approaches to predict active pathways and TF activities from single-cell RNA-seq data. Specifically, they evaluated the capacity of PROGENy and DoRothEA gene sets combined with the statistics implemented in the AUCell and VIPER tools to retrieve TF and pathway activities on in silico and in vitro sc-RNAseq data. Moreover, they assessed the capacity of these approaches to cluster distinct cell types on 13 scRNA-seq datasets (different protocols on the same cell types). The authors claim that tools developed for bulk functional genomics can be applied to scRNA-seq data, outperforming single cell tools. The study is of high interest to the community, the manuscript well written, and the analyses overall well performed.

*Response: We thank the reviewer for the positive comments.*

However, the analyses do not fully support the main claim of the paper, which should be at least soften.

*Response: The reviewer raises important and valid points that we have attempted to correct as outlined below.*

Specific comments are provided hereafter.

1. The main claim of the authors right from the abstract is that "bulk functional genomics tools can be applied to scRNA-seq data, outperforming dedicated single cell tools." However, their clearly state in the discussion that their analyses suggest that "the performance of functional genomics tools is more sensitive to the selection of gene sets than the statistical methods."

*Response: The reviewer raises a valid point. In retrospect, the phrase “bulk functional genomics tools can be applied to scRNA-seq data, outperforming dedicated single cell tools” was not precise enough.*

Any functional analysis tool uses a certain gene set and a certain statistical method. What we have found is that gene sets that are based on manually curated footprints (gene sets from PROGENy and DoRothEA) outperformed those that are purely predicted. In terms of the statistical method, we see some differences, and VIPER appears to perform better than AUCell.

*We have clarified this in the revised manuscript and softened the conclusion. In the abstract, instead of “bulk functional genomics tools can be applied to scRNA-seq data, outperforming dedicated single cell tools” we now state:

Our analyses suggest that bulk-based functional analysis tools that use manually-curated footprint gene sets can be applied to scRNA-seq data, partially outperforming dedicated single-cell tools. Furthermore, we found that the performance of functional analysis tools is more sensitive to the gene sets than to the statistic used.*

Hence, the comparisons performed by the authors might be biased due to the specific sets of genes they used (namely from DoRotheEA and PROGENy). This is confirmed by the fact that metaVIPER is performing poorly while VIPER on DoRothEA performs better. Hence, AUCell might perform better than VIPER on another gene set than DoRothEA.

*Response: The reviewer is right that we had focused on the specific sets from DoRotheEA and PROGENy and that we cannot be sure of what would happen with other gene sets. We now include SCENIC (see next point #2) into the benchmark expanding the collection of gene set resources. With SCENIC, and the tissue-specific GTeX regulons used with metaVIPER and DoRoThEA, we include a range of approaches from curated to purely inferred regulons.*

2. The authors claim that "high confidence TF regulons from DoRothEA outperform pure ARACNe regulons," emphasizing "the importance of combining literature curated resources with in silico predicted resources". It would be interesting to see how the SCENIC tool (relying on AUCell) performs on identifying key TFs involved in driving the different cell types and its capacity to cluster sc-RNAseq.

*Response: We thank the reviewer for this suggestion. Indeed it is very relevant to include SCENIC into our benchmark as this is a widely used tool in the community. We integrated SCENIC into the in vitro benchmark and into the analysis of PBMC data. With the in vitro benchmark we found that SCENIC was outperformed by tools using DoRothEA gene sets (DoRothEA and D-AUCell). With the PBMC data we found that TF activities predicted via SCENIC yielded a very good cluster purity, comparable to the top 2000 highly variable genes. Of note, the protocol specific SCENIC gene regulatory networks (e.g. Drop-seq specific regulons inferred from single-cells data profiled with Drop-seq) overlapped only in 24 TFs. This low overlap is not expected as all scRNA-seq protocols profiled the transcriptome of the same biological context so that we investigated possible functional consequences. We show that the TF activities predicted from SCENIC do not correlate well across the technologies/protocols. We added this analysis to the result section:*

*The low overlap of the SCENIC regulons motivated us to investigate the direct functional consequences of their usage. Theoretically, one would expect to retrieve highly similar regulons as they were constructed from the same biological context. We calculated the pairwise (Pearson) correlations of TF activities between the scRNA-technologies for each tool. The distribution of correlation coefficients for each tool denotes the consistency of predicted TF activity across the protocols (Fig. S10). The tools DoRothEA, D-AUCell and metaVIPER had all a similar median Pearson correlation coefficient of ~0.63, and SCENIC of 0.34. This suggests that the predicted TF activities via SCENIC networks are less consistent across the protocols than the TF activities predicted via DoRothEA, D-AUCell and metaVIPER.*


Fig. S10: Pairwise (Pearson) correlations of TF activities between the scRNA-technologies for each TF analysis tool.

*Response: In addition SCENIC regulons are constructed from and applied to the same dataset. Given this fact and the weak TF correlation across protocols/technologies we see indications that SCENIC’s good performance might be due to overfitting. We included this in the discussion.*

TF activities computed with DoRothEA, D-AUCell and SCENIC yielded a reasonable cluster purity. It should be noted that, while DoRothEA and D-AUCell rely on independent regulons, the SCENIC networks are constructed from the same dataset they are applied to. This poses the risk of overfitting. The fact that TF activities from SCENIC correlated across technologies worse than from the other tools is consistent with such an overfitting, but further analysis is required.

Indeed, in its associated publication, SCENIC is described as able to identify TF regulons automatically (without relying on prior knowledge such as DoRotheEA) and use this information to cluster cells.

*Response: Of note, indeed SCENIC is largely data driven but it does use some prior knowledge: the first step is purely data-driven, where basically co-expression modules (matching a regulator/TF to co-expressed genes) are identified by random forest. In the second step (RcisTarget) it is checked whether the genes within a co-expression module share the motif binding site of the corresponding regulator. This motif binding site information is extracted from databases and thus based on prior knowledge.*

The analyzed datasets and metrics used to assess the clustering capacity of SCENIC in its publication could be used for comparison with DoRothEA.

*Response: We agree with the reviewer that it is important to compare the cluster capacity of SCENIC with DoRothEA (and of course all other tools), which we do now in the revised version.

In the original SCENIC paper a mouse dataset was used to access the clustering potential. We focus on human datasets, for which the curated resources we have evaluated were developed. Applying them to mouse would inject additional noise to the evaluation. We hence believe that it is better to stick to the human PBMC dataset.

Regarding the different metrics, we used silhouette widths and the SCENIC authors used the adjusted rand index. While the adjusted rand index requires to run a clustering algorithm beforehand, silhouettes simply rely on distances in the reduced dimension space. Hence, silhouettes are independent of the various clustering algorithms that one could use and thus, we believe, a more generic metric than the adjusted rand index.*

As such, SCENIC goes one step beyond the combination of VIPER and DoRothEA as it predicts TF regulons and TF activities in single cells and uses it to cluster cell types.

*Response: As the reviewer points out, in the SCENIC article the authors perform clustering after the prediction of (binarized) TF activities for single cells. This is equally possible downstream of DoRothEA + VIPER. Both tools can and were combined in the manuscript with a downstream clustering analysis, investigating to which extend TF activities preserve cell type specific information.*

3. The title would need to be more specific as the evaluation was specific to TF and pathway activities estimation and not all "functional genomic tools".

*Response: We changed the title to

Robustness and applicability of transcription factor and pathway analysis tools on single-cell RNA-seq data

In addition we replaced the term “functional genomics tools” by “functional analysis tools” throughout the entire manuscript.*

Reviewer #2

Functional analysis of genomics data has greatly increased our understanding of our organs and tissues are specified and how they function. A large literature exists that describes the gene regulatory networks or pathway activity analysis from bulk tissue-specific RNA sequencing data.
Recently, there has been a revolution in technology that allows single cells to be profiled at the transcriptome level. Much as previously we have been able to understand the transcriptional difference between tissues, it is now becoming possible to examine the difference between individual cells. However, to really obtain insights from this single cell data a number of computational challenges need to be addressed. First and foremost is the sparsity of scRNAseq data. Due to our inability to capture all transcripts in a single cell, scRNAseq data has a very high drop-out rate where individual genes are not seen as being expressed primarily because of the stochasticity of message capture during the library preparation process. This has meant that different laboratories have started to develop algorithms that are specifically designed to analyse this scRNAseq data.

This manuscript compares whether these single cell specific algorithms perform better than the methods that have previously been developed for bulk data. This is a very timely and pertinent questions as the amount of scRNAseq data that is being generated is increasing at an astonishing pace.

The authors compare a number of algorithms that perform two distinct functions. Firstly algorithms that determine the activity of specific pathways, in particular the bulk analysis tools PROGENy and GO-enrichment are compared to AUCell, and secondly algorithms that build gene regulatory networks (DoRothEA with VIPER for bulk) is compared to single cell GRN methods such as metaVIPER. For their analysis the authors use either bulk, simulated scRNAseq data and real scRNAseq data. Their analyses are appropriate for the questions asked.

*Response: We thank the reviewer for her/his positive comments.*

Somewhat surprisingly to me, the authors find that the methods designed for bulk outperform those developed for single cells. For me also very interesting was the insight that the choice of gene sets used in these types of algorithms is more important than the choice of statistical method to assess enrichment of the particular gene set, and this could be highlighted in the abstract (Currently only mentioned in discussion).

*Response: We agree that this is an interesting insight, and we now included our finding/hypothesis that* *gene sets appear to be are more important for functional analysis than the statistics also in the abstract:

Our analyses suggest that bulk-based functional analysis tools that use manually curated footprint gene sets can be applied to scRNA-seq data, partially outperforming dedicated single-cell tools. Furthermore, we found that the performance of functional analysis tools is more sensitive to the gene sets than to the statistic used.*

Overall these conclusions are of interest to the rapidly growing single cell genomics community and deserve publication.

*Response: We are glad to hear the reviewers’ positive evaluation of our work.*

However, in a number of areas I would have liked to see additional analysis or further information. I have made some suggestions below. As it stands I found the paper very hard to follow.

*Response: We thank the reviewer for her/his valuable suggestions, that we believe have improved our manuscript.*

1. The authors analyse TF perturbation data that was previously published. The results of the global analysis are analysed with ROC and precision recall curves. Whilst I am sure this is a mathematically robust way of presenting the analysis, my gut feeling is that this may not tell the whole story. For example, I would imagine that some pathways are very robust and invariably recalled, even when drop-out rates are high, whilst other pathways may be much more sensitive, for example because this pathway is not very active in the cell type analysed. Could some extra information be provided to give a sense of this ? ( If my assumption of this variability is correct) The spread of results is given (Fig 1) but I cannot tell if it is the same pathways that are always at the top of that range.

*Response: We thank the reviewer for this interesting and relevant question, that we have addressed as follows:
To study the performance of individual pathways it is required to have a sufficient number of perturbation experiments per pathway. For the general robustness study we had several perturbation experiments per pathway. Hence, we evaluated PROGENy’s and GO-GSEA performance pathway-wise with respect to the number of footprint genes and gene coverage (AUROC and AUPRC). We found that the pathway-wise evaluation supports our initial conclusion that PROGENy recovers perturbed pathways better than GO-GSEA. Furthermore, this analysis also provided us insight into which pathway activity prediction is more and less reliable.

The result is available in the new Supplementary Figure 2


Fig. S2: Pathway-wise evaluation of a PROGENy and b GO-GSEA at different gene coverages. Performance is measured as Area under the ROC curve (AUROC). The dashed line indicates the performance of a random model. The colors in b are meant only as a visual support to distinguish between the individual volcano plots and jittered points.

and mentioned in the main text:

As our benchmark data set comprises multiple perturbation experiments per pathway we also evaluated the performance of PROGENy and GO-GSEA at the pathway level (Fig. S2a and b). The pathway-wise evaluation supported our finding that PROGENy outperforms GO-GSEA across all gene coverages.

Response: For the in-silico benchmark, however, we only had in total 15 pathway perturbation experiments for validation covering only 7 (out of 14) pathways. Hence, we have limited data to perform pathway wise evaluation.*

2. Minor point: do the colours in Fig 1a and c mean anything?

*Response: The colors in Fig. 1a and c do not have a meaning. They are meant as visual support to distinguish the volcano plots including the jittered point. We clarify this now in the figure legend.

Fig. 1: Testing the robustness of DoRothEA (AB), PROGENy and GO-GSEA against low gene coverage. a DoRothEA (AB) performance (Area under ROC curve, AUROC) versus gene coverage. b PROGENy performance (AUROC) for different number of footprint genes per pathway versus gene coverage. c Performance (AUROC) of GO-GSEA versus gene coverage. The dashed line indicates the performance of a random model. The colors in a and c are meant only as a visual support to distinguish between the individual volcano plots and jittered points.*


3. For their second analysis, (p8) the interest of the paper would be extended to a more generalist audience if this section briefly described and contrasted the key features of each of the methods in more detail.

*Response: We extended the method part and introduced each functional analysis tool, gene set resource and statistical method in greater detail:
Functional analysis tools, gene set resources and statistical methods
PROGENy: PROGENy is a tool that infers pathway activity for 14 signaling pathways (Androgen, Estrogen, EGFR, Hypoxia, JAK-STAT, MAPK, NFkB, PI3K, p53, TGFb, TNFa, Trail, VEGF and WNT) from gene expression data [33,40]. By default pathway activity inference is based on gene sets comprising the top 100 most responsive genes upon corresponding pathway perturbation, which we refer to as footprint genes of a pathway. Each footprint gene is assigned a weight denoting the strength and direction of regulation upon pathway perturbation. Pathway scores are computed by a weighted sum of the product from expression and the weight of footprint genes.

DoRothEA: DoRothEA is a gene set resource containing signed transcription factor (TF) - target interactions [13]. Those interactions were curated and collected from different types of evidence such as literature curated resources, ChIP-seq peaks, TF binding site motifs and interactions inferred directly from gene expression. Based on the number of supporting evidence each interaction is accompanied by an interaction confidence level ranging from A to E, with A being the most confidence interactions and E the least. In addition, a summary TF confidence level is assigned (also from A to E) which is derived from the leading confidence level of its interactions (e.g. a TF is assigned confidence level A if at least 10 targets have confidence level A as well). DoRothEA contains in total 470,711 interactions covering 1,396 TF targeting 20,238 unique genes. We use VIPER in combination with DoRothEA to estimate TF activities from gene expression data, as described in [13].

GO-GSEA: We define GO-GSEA as an analysis tool that couples GO-terms from MsigDB with the GSEA framework [7].

VIPER: VIPER is a statistical framework which was developed to estimate protein activity from gene expression data using enriched regulon analysis performed by the algorithm aREA [15]. It requires information about interactions (if possible signed) between a protein and its transcriptional targets and the likelihood of their interaction. If not further specified, this likelihood is set to 1. In the original workflow, this regulatory network was inferred from gene expression by the algorithm ARACNe providing mode of regulation and likelihood for each interaction [35]. However, it can be replaced by any other data resource reporting protein target interactions.

metaVIPER: metaVIPER is an extension of VIPER that uses multiple gene regulatory networks [19]. TF activities predicted with each individual gene regulatory network are finally integrated to a consensus TF activity score.

SCENIC: SCENIC is a computational workflow that predicts TF activities from scRNA-seq data [18]. Instead of interrogating predefined regulons, individual regulons are constructed from the scRNA-seq data. First TF-gene co-expression modules are defined in a data-driven manner with GENIE3. Subsequently those modules are refined via RcisTarget by keeping only those genes than contain the respective transcription factor binding motif. Once the regulons are constructed the method AUCell scores individual cells by assessing for each TF separately whether target genes are enriched in the top quantile of the cell signature.

D-AUCell/P-AUCell: The statistical method AUCell is not limited to SCENIC regulons. In principle it can be combined with any gene set resources. Thus, we coupled AUCell with gene sets from DoRothEA (D-AUCell) and PROGENy (P-AUCell). In comparison to other statistical methods AUCell does not include weights of the gene set members. Thus, the mode of regulation or the likelihood of TF-target interactions or weights of the PROGENy gene sets are not considered for the computation of TF and pathway activities.*


4. The next analysis is supposed to be illuminated by Fig s2. However, the figure legend and the labels are very uninformative. E.g. count: is this: counts per gene per cell?
Coverage? Experiment? This requires much more detail to be interpretable. This also applies to a number of other figures.

*Response: We apologize that the terms coverage, experiments and count were not well enough defined. Coverage denotes the number (count) of distinct perturbed pathways/TF in the benchmark data that are also covered by the gene set resource. E.g. an experiment where the pathway Nrf2 is perturbed would not contribute to the coverage (count) as this pathway is not covered within the PROGENy gene sets. However, a perturbation of the pathway MAPK would contribute. As there can be multiple independent perturbation experiments targeting a single pathway, e.g. MAPK, we also introduce the number of total perturbation experiments whose targets are covered by the gene set resource.
We clarified this in the legend:

Fig. S3: Overview of the benchmark dataset of the in silico study for a TF and b pathway analysis tools. The term coverage denotes the number of distinct perturbed TFs and pathways in the benchmark dataset covered by the respective gene set resource. As individual pathways/TFs can be perturbed several times in independent experiments we also provide the total number of perturbation experiments. In the case of TF perturbation experiments we also provide DoRothEA’s confidence class for each perturbed TF indicating the quality of its regulon within DoRothEA (A - high quality to E - low quality).

as well as in the main text:

We performed this procedure also for different TF confidence level combinations and show the performance change in these values in relation to the number of distinct perturbed TFs in the benchmark that are also covered by the gene set resources that we refer to as TF coverage (Fig. 2b - 2. step).

Response: In addition, we updated all figure legends with extended clarifications.*

5. The analysis described in Fig 2 is not clearly explained. E.g on page 9: Parameter combination (number of cells= 10, mean library size=5000). I found the terms used not self-explanatory and ill-defined.

*Response: We apologize for this lack of clarity. We clarified the definition of “number of cells” and “mean library size”:

We determined the total number of gene counts for a simulated single cell by sampling from a normal distribution with a mean equal to the desired library size which is specified as the first parameter of the simulation. We refer hereafter to this number as the library size. For every single cell, we then sampled with replacement genes from the gene probability vector up to the determined library size. The frequency of occurrence of individual genes becomes the new gene count in the single cell. The number of simulated single cells from a single bulk sample can be specified as the second parameter of the simulation. Of note, this parameter is not meant to reflect a realistic number of cells, but it is rather used to investigate the loss of information: the lower the number of simulated cells, the more information is lost from the original bulk sample (Fig. 2a; see Methods).*

Simulation of 10 cells seems to me to be very non-representative of any single cell data set (or are they referring to the number of times the simulation was carried out? ) Why was the number of 10 chosen for most of the analysis?

*Response: We thank the reviewer for this question. Indeed, the number of cells does not reflect a realistic number of cells in a single-cell data set. We chose it as we wanted to test the loss of information for up to an extreme case (since number of cells corresponds inversely to the loss of information). The lower the number of simulated cells, the more information is lost from the original bulk sample. We also performed the analysis for up 100 cells, where we see that even for such a low number of cells we reach the performance of bulk data (Fig. S7). We clarified this in the manuscript (see response above)*

Could the term mean library size be made clearer (total number of unique reads per cells or is it the total number of genes detected? )

*Response: We define the total number of read counts per sample/cell as the library size of a single sample/cell. Alternatively, the term sequencing depths could be used. We clarified its meaning in greater detail in the manuscript (see response above)*

6. The term TF coverage is used without explaining what this actually is. I therefore do not understand the analysis presented in figure 2b, step2.

*Response: Apologies, we explain the term TF coverage in greater detail in the revised version (see response to point #4).*

7. Step 3: "absolute difference"- this is not explained in the results.

*Response: We changed the term “absolute difference” to difference and explain its meaning in the manuscript:

Finally, we explored the effect of the simulation parameters library size and the number of cells on the performance by repeating the previously described analysis for all combinations of library sizes and cell numbers. We computed the mean difference between AUROC scores of single-cell and bulk data across all confidence level combinations. A negative difference indicates that the tool of interest performs overall better on bulk data than on scRNA-seq data, and a positive difference that it performs better on scRNA-seq. We observed a gradually decreasing negative difference approaching zero when the size of the library and the number of cells increase (Fig. 2b - 3. step and Fig. S7a). Of note, the number of cells and thus the amount of lost information of the original bulk sample has a stronger impact on the performance than the mean library size.*

8. In Fig 3 the most notable feature is that in two out of three panels the AUROC values were very low. Perturb-seq is a difficult experiment to carry out. Is this the most appropriate data set to test the performance of the algorithms on?

*Response: The reviewer raises a valid point. As mentioned in the discussion, we are aware of the issues related to the Perturb-seq dataset. However, TF perturbation data on single cell level are sparse and there are not many alternative datasets. Hence, we decided to use the few datasets available, while remaining conscious of their limitations. These limitations was also a major motivation to perform the simulation and the complementary clustering study.*

Whilst the authors state that the ground truth is known in these experiments, I wonder whether this is really the case.

*Response: The reviewer is right that the ground truth is not truly known in general. This is indeed a limitation of such benchmark exercises. We had mentioned the specific issue of the error-prone matching of sgRNA and cell ID, and we now acknowledge this more generally in the discussion:

In addition, our definition of true-positives and true-negatives is commonly used for such analyses [4,13,37], but it might be incorrect due to indirect and compensatory mechanisms [39]. These phenomena can confound the results of this type of benchmarks.*

9. The final analysis shows some very interesting trends, especially the good cluster separation when using TF activity rather than TF expression, although even TF activity performed less well than to top HVG (Fig 4b). Overall the figure highlights the value of directionality (ie activation or repression) within the algorithms that assesses the TF activity. Given that DoRothEA makes specific predictions about which TFs are likely to be essential for each of these cell types, can the authors validate any of their conclusions. Can they either generate this data or compare it to data that is out there? E.g TF essentiality data may be of use here; or TF dependencies in cancer data.

*Response: We thank the reviewer for the interesting question. We integrated a new analysis showing that TF activity correlates, albeit weakly, with TF essentiality (see the new Supplementary Figure 15). The findings are described in a new paragraph.

Besides these individual examples, we analyzed the biological relevance of the identified TF activities in more detail. We assumed that the highly active TFs are regulating important cellular functions, resulting in a correlation between TF activity and essentiality. As (to our knowledge) no gene essentiality data is available for PBMCs, we used hematologic cancer (lymphoma and leukemia) gene essentiality data from the DepMap project [31]. We compared the difference between the TF activities in lymphoid (B, T and NK cells) and myeloid (monocytes and dendritic cells) PBMCs with the TF gene essentiality differences between myeloid and lymphoid hematologic cancers. SPI1, according to its higher activity in myeloid PBMCs, was more essential in myeloid leukemias (Fig. S15a and b, Wilcoxon-test p-value = 0.038). For a more comprehensive analysis, we compared the differences in TF activity (PBMCs, lymphoid - myeloid) and the differences in TF gene essentiality (hematologic cancers, lymphoid - myeloid) by calculating their Pearson correlation for all TFs. The TF activities predicted by DoRothEA correlated best with the respective essentiality scores across all scRNA-seq protocols (median Pearson correlation coefficient of 0.107; 0.08 for D-AUCell; 0.04 for metaVIPER and -0.002 for SCENIC, Fig. S15c). The difference in TF activities predicted with DoRothEA from the dataset generated by Smart-Seq2 and Quartz-Seq2 correlated significantly with the difference in essentiality (Pearson correlation, p-value of 0.049 and 0.032, respectively). Thus, TF activities predicted with DoRothEA regulons correlate, weakly but more than the activities predicted with other tools, with gene/TF essentiality.*


Fig. S15: a TF activity and b gene essentiality for the TF/gene SPI1 in lymphoid and myeloid cells. c Correlation of the differences in TF activity and differences in gene essentiality separately for all tool-protocol combinations. The results are summarized for each TF analysis tool. The points on the volcano plots represent the different scRNA-seq protocols.

In conclusion, I feel the authors have a very interesting story to tell.

*Response: We thank the reviewer for the positive comment.*

However, more background info on the analysis that are carried out would make this paper much more accessible. Figure legends and precise definitions of terms used could be greatly improved.

*Response: We hope the revised version has improved these aspects, and we thank the reviewer for the feedback that helped us improve the manuscript.*

Minor errors/typos:

page 5: Specifically, we (no capital)

*Response: Done*

page 17; line 415- that should be than

*Response: Done*

**Second round of review**

**Reviewer 1**

I thank the authors for all the added work in this revised version and for addressing all my comments. This is a very nice piece of work that I am looking forward to seeing published.

**Reviewer 2**

The authors carried out significant additional analysis to address the comments and I believe the paper is now much improved.

A few specific comments:
Line 179; While I agree with the statement that “PROGENy outperforms GO-GSEA across all gene coverages.” It is also clear that the performance is very variable between pathways. Maybe the authors could soften the statement to something like: PROGENy outperforms GO-GSEA across all gene converages, but performance between gene signatures is variable.

Please note that in a number of places (Fig 1, S2, S15) violin plots are referred to as volcano plots. Usually the term volcano plots refers to differential gene expression analysis plot (significance versus fold change).

Minor comments. As I read through I noticed some unclear sentences and have suggested some modifications to improve these: (add underlined text)

1. It is thus not clear if functional TF network and pathway analysis tools established for bulk sequencing can be applied to scRNA-seq in a meaningful way.

2. However, they still have a number of limitations and biases, including low library size, and drop-outs.
(“They” refers to technologies and protocols)
However, single cell data sets still have a number of limitations and biases, including low library size, and drop-outs.

3. Figure 1, S2 and Figure S15: Do you mean violin rather than volcano plots?

4. We reason that this observation might be due to the quality of the corresponding benchmark data

5. line 732; The fact that TF activities from SCENIC correlated across technologies worse than from the other tools is consistent with such an overfitting.
change to:
Across technologies, the TF activities from SCENIC correlated less well than those calculated with the other tools, which is consistent with overfitting by SCENIC.

6. line 746; …partially outperforming partially tools specifically designed for scRNA seq analysis.

**Authors Response**

**Point-by-point responses to the reviewers’ comments:**

The authors carried out significant additional analysis to address the comments and I believe the paper is now much improved.

*We thank the reviewer for the positive comment.*

A few specific comments:

Line 179; While I agree with the statement that “PROGENy outperforms GO-GSEA across all gene coverages.” It is also clear that the performance is very variable between pathways. Maybe the authors could soften the statement to something like: PROGENy outperforms GO-GSEA across all gene converages, but performance between gene signatures is variable.

*Response: Based on the reviewer’s legitimate comment we softened the conclusion as follows:*

*The pathway-wise evaluation supported our finding that PROGENy outperforms GO-GSEA across all gene coverages, but the performance between pathways is variable.*

Minor comments. As I read through I noticed some unclear sentences and have suggested some modifications to improve these: (add underlined text)

*Response: We thank the reviewer for the attentive comments to our manuscript*

1. It is thus not clear if functional TF network and pathway analysis tools established for bulk sequencing can be applied to scRNA-seq in a meaningful way.

*Response: We thank the reviewer for this suggestion. Since we are limited in the number of words in the abstract, we have reworded the sentence as follows:*

*It is thus not clear if functional TF and pathway analysis tools established for bulk sequencing can be applied to scRNA-seq in a meaningful way.*

2. However, they still have a number of limitations and biases, including low library size, and drop-outs.

(“They” refers to technologies and protocols)

However, single cell data sets still have a number of limitations and biases, including low library size, and drop-outs.

*Response: The reviewer is right. We updated the sentence according to the reviewer’s suggestion:*

*However, single-cell data sets have a number of limitations and biases, including low library size, and drop-outs.*

3. Figure 1, S2 and Figure S15: Do you mean violin rather than volcano plots?

*Response: We thank the reviewer for this attentive comment. Indeed we mixed up the terms “volcano” and “violin” plots. We have corrected this error in the entire manuscript and supplementary documents.*

4. We reason that this observation might be due to the quality of the corresponding benchmark data

*Response: We updated the sentence according to the reviewer’s suggestion:*

*We reason that this observation might be due to the quality of the corresponding benchmark data*

5. line 732; The fact that TF activities from SCENIC correlated across technologies worse than from the other tools is consistent with such an overfitting.

change to:

Across technologies, the TF activities from SCENIC correlated less well than those calculated with the other tools, which is consistent with overfitting by SCENIC.

*Response: We updated the sentence according to the reviewer’s suggestion:*

*Across technologies, the TF activities from SCENIC correlated less well than those calculated with the other tools, which is consistent with overfitting by SCENIC, but further analysis is required.*

6. line 746; …partially outperforming ~~partially~~ tools specifically designed for scRNA seq analysis.

*Response: We updated the sentence according to the reviewer’s suggestion:*

*Our systematic and comprehensive benchmark study suggests that functional analysis tools that rely on manually curated footprint gene sets are effective in inferring TF and pathway activity from scRNA-seq data, partially outperforming tools specifically designed for scRNA-seq analysis.*
